# On the Perception of Moral Standing to Blame

**DOI:** 10.1162/opmi_a_00185

**Published:** 2025-01-20

**Authors:** Isaias Ghezae, Fan Yang, Hongbo Yu

**Affiliations:** Department of Psychological and Brain Sciences, University of California, Santa Barbara, Santa Barbara, CA, USA; Department of Psychology, The University of Chicago, Chicago, IL, USA

**Keywords:** blame, moral standing, moral commitment, moral knowledge

## Abstract

Is everyone equally justified in blaming another’s moral transgression? Across five studies (four pre-registered; total *N* = 1,316 American participants), we investigated the perception of *moral standing to blame*—the appropriateness and legitimacy for someone to blame a moral wrongdoing. We propose and provide evidence for a moral commitment hypothesis—a blamer is perceived to have low moral standing to blame a moral transgressor if the blamer demonstrates weak commitment to that moral rule. As hypothesized, we found that when blamers did not have the chance or relevant experience to demonstrate good commitment to a moral rule, participants generally believed that they had high moral standing to blame. However, when a blamer demonstrated bad commitment to a moral rule in their past behaviors, participants consistently granted the blamer low moral standing to blame. Low moral standing to blame was generally associated with perceiving the blame to be less effective and less likely to be accepted. Moreover, indirectly demonstrating moral commitment, such as acknowledging one’s past wrongdoing and feeling/expressing guilt, modestly restored moral standing to blame. Our studies demonstrate moral commitment as a key mechanism for determining moral standing to blame and emphasize the importance of considering a blamer’s moral standing as a crucial factor in fully understanding the psychology of blame.

## INTRODUCTION

Blame is an important mechanism for maintaining successful cooperation and functioning in societies (Malle et al., 2013, 2022; Sher, 2006; Tognazzini & Coates, 2024). It is commonly defined as a speech-act^1^ designed to morally condemn other agents (individuals, groups, corporations, etc.), with the aim of calling out behaviors and/or attitudes that violate certain moral norms and thereby enforcing these norms (Brunning & Milam, 2018; Mason, 2019; Sher, 2006; Tognazzini & Coates, 2024). As such, blame typically involves three elements—the target of the blame (i.e., blamee), the behavior (broadly construed, including but not limited to, an action, omission, attitude, and emotion) of the blamee that violates the moral norm in question, and a blamer who does the blaming. Extensive psychological research and theory on blame has revealed how various characteristics and actions of the blamee justify the appropriateness of blame (Anderson et al., 2020; Malle, 2021; Malle et al., 2014, 2022). However, this literature remains relatively silent regarding the conditions of the *blamer* that are relevant to the moral status, effectiveness, and reception of the blame. Does everyone have equal legitimacy to blame an instance of a moral violation? Do we view some people as having less moral standing to blame than others? In this paper, we systematically examine perceptions of moral standing to blame and demonstrate that an individual’s moral commitment is a key mechanism underlying an individual’s moral standing to blame others.

### Moral Standing to Blame and Related Concepts

Evaluating an instance of blame is different from evaluating blameworthiness, with the latter having been extensively studied in social psychology and philosophy (Malle, 2021; Mason, 2019; Scanlon, 2008; Sher, 2006; Smith, 2007; Wallace, 2010). In an act of blame, the blamer “must demand uptake from the addressee” (i.e., blamee) (Lippert-Rasmussen, 2023). In other words, by blaming the blamee, the blamer requires the blamee to take the blame seriously and to conform to it, usually against the blamee’s own will or wish. Therefore, moral authority of some sort would be expected of the blamer. In the philosophical literature, the moral authority expected of the blamer in order for their blame to be taken seriously by the blamee is termed *moral standing to blame* (Lippert-Rasmussen, 2023; Todd, 2019).

Relevant to but distinct from the concept of moral standing, past research has examined the concept of psychological standing (Effron & Miller, 2015; Miller & Effron, 2010; Oc & Kouchaki, 2024), focusing on how an agent’s psychological characteristics, such as their social identities and past suffering affect their legitimacy to speak up and take actions for certain issues. For example, citizens of a country are perceived to be more appropriate and legitimate critics of the country’s policies and history than non-citizens (Hornsey & Imani, 2004). People who are negatively impacted by a policy and suffer from it have more psychological standing to criticize and protest against it (Effron & Miller, 2015; Ratner & Miller, 2001).

In contrast to this line of research, our focus here is on *moral standing to blame* (Lippert-Rasmussen, 2023; Todd, 2019). While both psychological standing and moral standing concern issues of legitimacy, psychological standing emphasizes psychological characteristics—such as identities and experiences of suffering—as sources of perceived legitimacy. In contrast, moral standing centers on relevant moral characteristics, including moral knowledge, behaviors, and experiences, as the basis for legitimacy. Compared with social identities and past suffering, *moral* standing may have greater potential for voluntary changes and improvement, yet it remains underexplored in the literature. Therefore, by investigating moral standing to blame, we can broaden the scope of empirical research on sources of standing and legitimacy.

### Does Everyone Have Equal Standing to Blame?

At first sight it may seem clear that when evaluating an instance of blame, one should only consider the content of the blame (i.e., factual accuracy, appropriate degree), rather than any facts about the blamer. After all, if a certain act of blame accurately and appropriately points out a violation of common moral rules and norms, then all instances of said blame should be equally valid, and the blamee should have an equal obligation to accept the blame, regardless of who delivers it. In certain non-moral domains, the evaluation of the product of an agent can (or even should) be completely detached from the agent. For example, in peerreview processes of a scientific manuscript, the identity of the authors is often redacted. Even when the reviewers know the identity of the authors, they are not supposed to be influenced by such information. The object of evaluation in this case is the merit of the scientific research (i.e., the product), not the characteristics of the researchers. If the blame process was similar to the peerreview process, then what would matter would be the content of the blame (i.e., analogous to the scientific merit of a scientific manuscript), rather than the blamer (i.e., analogous to the identity of the authors of a scientific manuscript).

On the other hand, it has also been theorized that not everyone would have the same standing to blame. While existing psychology research and theories have largely overlooked how a blamer’s characteristics may affect their moral standing to blame, recent debates about the idea of moral standing to blame have been an active area in moral philosophy (Cohen, 2006; Edwards, 2019; Fritz, 2019; Fritz & Miller, 2018, 2019; King, 2020; Lacey & Pickard, 2021; Leibowitz, 2016; Lippert-Rasmussen, 2013, 2020; Piovarchy, 2020; Roadevin, 2018; Rossi, 2021; Statman, 2023; Todd, 2019). For example, such work has highlighted various scenarios in which “a speaker is ‘not in a position to’ lodge a certain moral criticism against another, in spite of the fact that the criticism is accurate and correct” (Dworkin, 2000, p. 184). Additional work has argued that merely uttering valid moral statements is insufficient for a moral criticism to be effective; the efficacy of blame also hinges on certain facts about the blamer that are relevant to its success (Cohen, 2006; Leibowitz, 2016). For example, it is legitimate for an individual to take a piece of moral advice (e.g., paying carbon taxes, donating to effective charities, practicing veganism) less seriously if the individual knows that the person who gives the advice does not practice what they preach (Leibowitz, 2016; Statman, 2023). Despite the extensive philosophical theorizing on this topic, there lacks analogous empirical work exploring these perceptions and, as such, it remains unknown whether laypeople’s moral judgments are consistent with these perspectives.

### Moral Commitment as a Mechanism of Moral Standing to Blame

Based on the philosophical theorizing summarized above, our primary hypothesis is whether a blamer having strong *moral commitment* to a moral rule is a critical mechanism for the perception of the blamer’s moral standing to blame (*moral commitment hypothesis*). By moral commitment, we primarily refer to someone’s commitment demonstrated in their outward behaviors and expressed attitudes.

One direct indicator for such commitment is previous behavior of the blamer—it seems inappropriate for a blamer to blame someone else for a wrongdoing that they themselves have committed. Indeed, a large volume of philosophical debates on moral standing to blame has focused on this condition (e.g., Darwall, 2006; Lippert-Rasmussen, 2023; Radzik, 2011; Todd, 2019). Blaming someone else for a wrongdoing that one has committed in the past is also a prototypical case of moral hypocrisy. Extensive research has demonstrated that people dislike hypocrites (Barden et al., 2005; Effron & Miller, 2015; Yu et al., 2022) who falsely signal moral superiority that they do not deserve (Jordan et al., 2017). However, little research has investigated how hypocrisy may affect a blamer’s standing to blame. Disliking hypocrites does not necessarily mean that one must disregard their advice if the advice is factually accurate and helpful. For example, one need not dismiss a doctor’s diagnosis or prescription on the grounds of the doctor’s likability. The question is, assuming that the content of the blame is factually accurate, do people perceive the blame from a hypocritical blamer as less legitimate, and therefore feel more justified to dismiss it?

If moral commitment matters for a blamer’s moral standing to blame, it would also be informative to know whether only committing the same moral violations as the blamee affects the blamer’s moral standing. Committing a qualitatively different but equally severe moral violation as the blamee, for example, may lead both the blamee and the observer to question the moral character and motives of the blamer (Brambilla et al., 2021; Luttrell et al., 2022; Siegel et al., 2018) and to suspect that the blamer would commit the same moral wrongdoing as the blamee if given the chance. Therefore, we hypothesized that the severity of the moral violation matters more than the type of the moral violation—violating a different moral rule as the blamee would reduce the blamer’s moral standing to blame, as long as the blamer’s moral violation is morally equally bad and severe as the blamee’s (the *severity hypothesis*).

We also acknowledge that, in reality, almost everyone has committed some wrongdoing at some point in their life. If past wrongdoing indeed reduces a blamer’s moral standing to blame, it would be theoretically and practically important to understand the mechanisms through which past moral wrongdoers could regain their standing to blame others. Without such a mechanism, our social and moral interactions would be unimaginable because hardly anyone would have the moral standing to blame others (Bell, 2013). Conceivably, indirect ways to demonstrate moral commitment to the moral rule, such as acknowledging one’s past wrongdoing and feeling guilt might help the blamer to regain moral standing (i.e., the *regaining standing hypothesis*).

### Moral Knowledge as a Potential Modulator

In real life, a blamer may blame moral violations that may or may not apply to himself or herself. When the moral rule applies to the blamer, moral commitment could be inferred from relevant past behaviors of the blamer. However, when the moral rule does not apply to the blamer, evaluating the moral commitment of the blamer would not be straightforward. In nonmoral domains, such as doctors giving medical advice, the doctors themselves may not have the illness they provide advice for, but they have good standing to give advice based on their medical knowledge and credentials (derived from relevant education and experience). Is it possible that the perception of moral standing to blame may also be influenced by the perception of the blamer’s moral knowledge and experience? As a secondary goal of the present research, we aimed to test the hypothesis that moral knowledge and experience related to a moral rule influences a blamer’s moral standing to blame, especially when the moral rule is not directly applicable to the blamer (the *moral knowledge*/*experience hypothesis*).

### The Present Research

Our research aimed to examine the mechanisms and effects of moral standing to blame. In particular, we focused on the blamer’s moral commitment to the moral rule as a key mechanism, especially when the moral rule directly applies to the blamer. We also explore whether the blamee and observers may want to know if the blamer has the necessary moral knowledge or experience to have the authority to comment on this issue, in instances where the moral rule does not apply to the blamer.

In five studies (four pre-registered), we investigated the effects of a blamer’s commitment to a moral rule on the perception of moral standing to blame. In four of these studies, the blamer’s moral knowledge/experience with regard to the moral issues they blame others for also varied. In Study 1, we developed and validated a measure of the perception of moral standing to blame and examined the joint effects of blamer’s moral commitment and relevant moral knowledge/experience on the perception of their moral standing to blame. Studies 2 and 3 directly examined the causal effects of blamer’s relevant moral knowledge/experience on their moral standing to blame by manipulating whether the blamer has faced the same moral issues that they blame the blamee for violating. The two studies differed in terms of the moral issues involved—while Study 2 adopted moral issues that are straightforward (e.g., cheating on one’s romantic partner), Study 3 adopted more complicated issues (e.g., stealing to feed one’s family members). Study 4 more closely compared moral standing, standing derived from the blamer’s moral characteristics (e.g., past moral behaviors, moral beliefs, etc.), with psychological standing, standing based on a blamer’s social identity or group membership (e.g., being a vegan). Finally, Study 5 tested the hypothesis that addressing one’s past wrongdoing, by acknowledging it and feeling guilty about it, would help a blamer to restore moral standing to blame others for the same wrongdoing.

All the studies adopted a between-subject design, where participants were randomly assigned to one of several conditions. Except for Study 1, the experimental protocols were preregistered. All cell sizes were larger than 50, which aligns with the recommendation by statisticians (Simmons et al., 2013). Data, analysis codes, and study materials for all studies are available at the OSF link: https://osf.io/68we2/.

## STUDY 1

As an initial exploration, our primary goals of Study 1 were to (1) develop a measure of moral standing to blame, (2) investigate the effects of a blamer’s demonstrated (lack of) moral commitment to a moral rule and blamer’s experience/knowledge of the moral rule on (2a) observers’ perceptions of the blamer’s moral standing to blame and (2b) the effectiveness and consequences of the blame. Based on the philosophical conjectures of the antecedents and consequences of moral standing to blame (Fritz & Miller, 2018, 2019; Todd, 2019; Tognazzini & Coates, 2024; Wallace, 2010), we hypothesized that: (1) the blamer’s demonstrated moral commitment to the moral rule they blame others for violating will have a positive impact on the perceived moral standing to blame (i.e., the *moral commitment hypothesis*); (2) as long as the blamer’s past wrongdoing is comparable to the blamee’s wrongdoing in severity (i.e., blameworthiness), whether the blamer violated the same or a different moral rule as the blamee will not matter for the perception of the blamer’s moral standing to blame (i.e., the *severity hypothesis*).

### Materials and Methods

#### Participants.

We recruited participants online using Prolific Academic (https://prolific.co/) for all the studies reported in this paper. A sensitivity power analysis indicated that to detect a medium effect (f = 0.25) in ANOVA with 95% power, we needed a total sample of 210 participants (roughly 53 for each group). Two hundred twenty-eight participants were recruited, 15 of whom were excluded due to failing our attention check questions, leaving us with a final sample of 213 participants (122 or 57.3% were females, *M*_*age*_ = 31.4, *SD*_*age*_ = 11.2). Here and in the subsequent studies, we also measured participants’ socioeconomic status using the standard ladder scale (Adler et al., 2000).

#### Experimental Design and Materials.

To test these predictions, we presented participants with vignettes and asked them to evaluate target characters in the vignettes. We operationalized rule relevance through membership in a particular social role or occupation; those who belonged to the same social role/occupation were considered to be governed by the same moral rule. In a 2 × 2 between-subjects design, we manipulated the blamer’s past moral behavior (immoral vs. morally neutral) and whether or not the blamer had the same social role as the blamee they blamed.

Participants were asked to read a short story about two individuals. Each vignette had one individual commit a moral transgression that was related to their social role (e.g., a chef who carelessly fails to abide by a customer’s specific request on an order thus causing outrage). The individual who committed the moral transgression (i.e., blamee) always received blame from the other individual in the vignette (i.e., blamer). To illustrate the experimental design, we present the four versions of a vignette we used. The complete vignettes can be found in the Supplementary Materials. Here, the blamee is a chef who ignores a customer’s order as requested:

**Ryan** is a chef at a popular restaurant when, one night, a customer orders the restaurant’s signature dish. The customer requests that the dish is made with very little salt. As **Ryan** prepares the dish, he pays no attention to the amount of salt he puts into the dish because he usually doesn’t care much for the specific requests of customers. **Ryan** finishes making the dish with much more salt than the customer requested. Almost immediately after consuming the food, the customer becomes angry and makes a huge complaint to the manager of the restaurant.

In the Immoral-Same role condition, the vignette continued,

The following day, **Ryan** recounts the story to **Derek** who is also a chef at the same restaurant. **Derek** has made the same mistake in the past where he carelessly failed to follow the specific request of a customer and made the customer upset.

In the Neutral-Same role condition, the vignette instead continued,

The following day, **Ryan** recounts the story to **Derek** who is also a chef at the same restaurant. **Derek** recalls once having received a similar request in the past. Furthermore, **Derek** remembers working carefully to ensure that the dish did not contain a lot of salt so that the customer would be satisfied.

In the two Different role conditions, the blamer is the headwaiter at the same restaurant, who is either always rude towards customers (Immoral condition) or works hard to make sure customers are satisfied (Neutral condition). All the four version then end with the blamer blaming the blamee:

During their conversation, **Derek** instantly blames **Ryan** for not caring enough about the customer which he claims is a basic requirement for being a chef.

Each participant was presented with one of the four conditions and were then asked to complete our set of measures.

### Measures

#### Moral Standing to Blame.

Since there is no existing empirical study on moral standing to blame, we developed a measurement of this concept by compiling a list of phrases that authors used to refer to it in philosophical literature. Specifically, participants evaluated the following five items on a continuous slider scale (0 = *not at all*, 100 = *extremely*):

- To what extent do you think it is legitimate for [blamer’s name] to blame [blamee’s name]?
- To what extent do you think it is appropriate for [blamer’s name] to blame [blamee’s name]?
- To what extent do you think [blamer’s name] is the right person to blame [blamee’s name]?
- To what extent do you think [blamer’s name] is in the position to blame [blamee’s name]?^2^
- To what extent do you think the relationship between [blamer’s name] and [blamee’s name] makes it [blamer’s name]’s business to blame Ryan?

Internal reliability for these items was good overall (Cronbach’s *α* = 0.89). Cronbach’s *α* for separate conditions were good for three conditions (Cronbach’s *α* between 0.86 and 0.90) and acceptable for one condition (*α* = 0.66). In the following analysis, we averaged these items into one composite score of moral standing to blame.

#### Effectiveness of Blame.

Participants were asked to rate how effective they perceived the blame to be. Specifically, participants evaluated the two items on a continuous slider (0 = *not at all*, 100 = *extremely*):

- How effective could [blamer’s name] blame towards [blamee’s name] be?
- How convincing could [blamer’s name] blame towards [blamee’s name] be?

Since these two items had good reliability across all four conditions (Spearman-Brown coefficient was used since there were only two items (Eisinga et al., 2013); overall *r* = 0.86, *r* for each condition ranged between 0.78 and 0.89), we averaged these two items together as one measure for the perceived effectiveness of blame.

#### Acceptance of Blame.

Participants were asked to indicate how much they would accept the blame if they were the blamee in the scenario. For this item, participants were asked, “If you were [blamee’s name], to what extent would you accept [blamer’s name] blame?” Participants answered this item using a continuous slider (0 = *not at all*, 100 = *completely*).

#### Improvement.

Participants were asked to indicate how much they think themselves would improve their behaviors after hearing the blamer’s blame if they were the blamee in the scenario. For this item, participants were asked, “If you were [blamee’s name], how likely would you do better the next time you have the opportunity to [avoid described moral transgression]?” Participants answered this item using a continuous slider (0 = *not at all*, 100 = *extremely*).

#### Self-Conscious Emotions.

Participants were asked to indicate their perceived self-conscious emotions if they were the blamee in the scenario. Specifically, they were asked, “If you were [blamee’s name], to what extent would [blamer’s name] make you feel the following emotions for [committing the described moral transgression]?” Participants used a continuous slider scale to rate five possible emotions: guilty, remorseful, anxious, ashamed, and embarrassed (0 = *not at all*, 100 = *extremely*). There was high internal reliability between the responses for each emotion across all four conditions (Cronbach’s *α* between 0.86 and 0.91) so we averaged these items into one composite score for self-conscious emotions.

For the following studies, we reused all the same measures for the dependent variables listed in Study 1.

### Results

#### Manipulation Checks and Preliminary Analysis.

To validate our manipulation of blamer’s past moral behavior and the blamer’s role/occupation, we included two manipulation check questions—to what extend the blamer does a good job in their own role, and to what extend the blamer is expected to perform the blamee’s job well. We expected that the participants assigned to the morally neutral conditions should report higher value for the first question than the participants assigned to the morally problematic conditions. Similarly, we expected that the participants assigned to the same role conditions should reported higher value for the second question than the participants assigned to the different role conditions. Both effects were significant as we expected (*F*(1, 208) = 169.90, *p* < 0.001 for the former, *F*(1, 208) = 52.08, *p* < 0.001 for the latter). These results indicated that the manipulations of blamer’s past moral behavior and the blamer’s role were successful.

The blamee’s behavior was seen more as a matter of morality than a matter of ability across all four conditions (*M* ± *SE* = −19.85 ± 2.23, where −50 = *A matter of morality*, 50 = *A matter of ability*). The blamee’s behavior was perceived to be morally blameworthy across all the four conditions (mean blameworthiness rating ranged from 77 to 87 on a scale of 0–100, where 100 = extremely blameworthy). These results confirmed that our participants did perceive the blamee’s behavior as a violation of morality. There was no significant difference in blameworthiness judgments across conditions (*F*(3, 209) = 2.29, *p* = 0.079), indicating that the differences in our interested variables (e.g., moral standing to blame, effectiveness of blame etc.) across conditions could not be attributed to differences in the blameworthiness of the blamee’s behavior. Similarly, the blamee was perceived to not fulfill the responsibility required by their role (mean fulfillment rating ranges from 21 to 28 on a scale of 0–100, where 0 = not at all). Again, there was no significant difference in fulfillment judgments across conditions (*F*(3, 209) = 1.06, *p* = 0.366).

#### Moral Standing to Blame.

**Figure 1.**
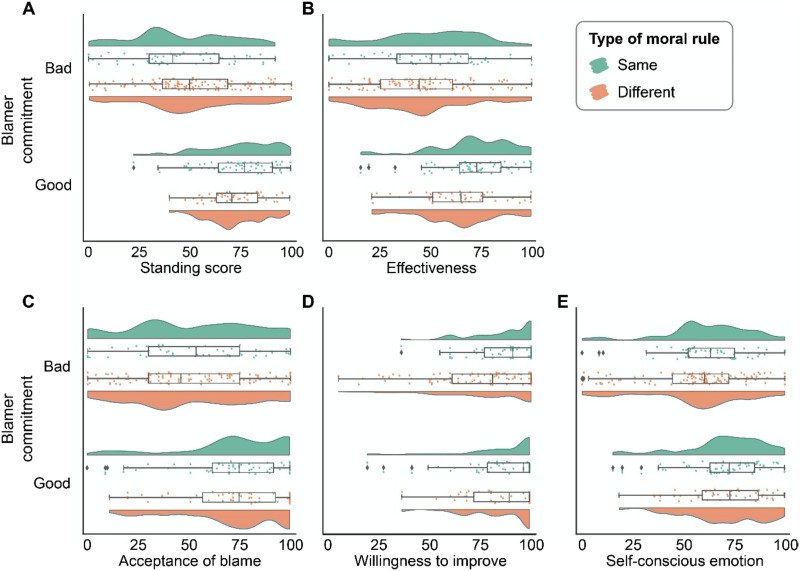
Results of Study 1. Primary dependent variables as a function of the moral commitment factor and the type of rule factor.

To address the moral commitment hypothesis, we ran a linear model to examine the effect of blamer’s moral commitment on the perception of the blamer’s moral standing to blame (Figure 1A). This model only included the Same role conditions because the moral commitment hypothesis is specifically about the blamer who demonstrates good or bad commitment to the same moral rule that the blamer blames others for violating. In this model, the composite score of moral standing to blame was treated as the dependent variable, the blamer’s moral commitment to the moral rule (good vs. bad) was included as a predictor. We also included participants age, gender, and socioeconomic status as covariates (excluding these covariates does not change the statistical results; Table S1). Supporting the moral commitment hypothesis, when the blamer demonstrated bad moral commitment to the same moral rule that they blamed the blamee for, the blamer was perceived to have lower moral standing to blame than when the blamer demonstrated good moral commitment to that rule (*B* = −29.69 ± 4.16, *b* = −1.15, *t* = −7.15, *p* < 0.001, 95% CI = [−37.93, −21.45]).

To test the severity hypothesis, we ran another linear model where we included all the four conditions. Again, the composite score of moral standing to blame was treated as the dependent variable. In this model, we included the main effect of type of rule (i.e., same vs. different moral rule) as the predictor. If the severity hypothesis is correct, then the main effect of type of rule should not be significant. This is indeed what we found: the main effect of type of rule was negligible (*B* = 1.74 ± 3.40, *b* = 0.07, *t* = 0.511, *p* = 0.610, 95% CI = [−4.96, 8.43]).

To more directly compare the effect size of the moral commitment factor (good vs. bad) and the type of rule factor (same vs. different moral rule), we ran a third model, which also included all four conditions. In this model, we included the main effect of commitment as the predictor. This model revealed a significant and large main effect of commitment, such that the blamer who demonstrated bad moral commitment in their past behavior, regardless of the type of moral rule, was perceived to have lower moral standing to blame than the blamer who demonstrated good moral commitment (*B* = −23.91 ± 2.97, *b* = −0.98, *t* = −8.05, *p* < 0.001, 95% CI = [−29.76, −18.05]). The confidence interval of the main effect of commitment had no overlap with that of the main effect of the type of rule, indicating that the former effect is stronger than the latter. This comparison lent further support for the severity hypothesis, namely, as long as the blameworthiness of the blamer’s past wrongdoing is comparable to that of the blamee’s, whether the blamer violates the same or different moral rule as the blamee does not matter too much for the perception of the blamer’s moral standing to blame.

We next explore if there was interaction between the two factors. In the fourth model, we included both the main effect of commitment and type of rule, and their interaction as the predictors. This model revealed a small but significant interaction effect (*B* = 11.84 ± 5.84, *b* = 0.49, *t* = 2.03, *p* = 0.044, 95% CI = [0.32, 23.35]). This interaction indicated a small moderation of the type of rule on the effect of commitment, such that blamer’s good versus bad moral commitment to a different rule, while still matter a lot for the perception of the blamer’s moral standing to blame (*B* = 17.71 ± 4.28, *b* = 0.789, *t* = 4.14, *p* < 0.001, 95% CI = [9.22, 26.21]), has a relatively smaller effect compared with blamer’s good versus bad moral commitment to the same rule (Figure 1A). Including the blameworthiness of the blamee’s behavior did not change the patterns of the results (Table S2).

#### Effectiveness of Blame.

We next examined the effects of blamer’s moral past and role on the perceived effectiveness of blame (Figure 1B). Not surprisingly and in line with philosophical conjectures (Tognazzini & Coates, 2024), blame from a blamer who demonstrated good moral commitment was perceived to be more effective than blame from a blamer who demonstrated bad moral commitment (*B* = 13.88 ± 4.52, *b* = 0.57, *t* = 3.07, *p* = 0.002, 95% CI = [4.96, 22.79]). Similar to the pattern of moral standing to blame, the type of rule had no impact on the perception of the effectiveness of the blame (*B* = −2.00 ± 4.45, *b* = −0.08, *t* = −0.45, *p* = 0.654, 95% CI = [−10.77, 6.77]). The interaction between the two factors was not significant (*B* = 8.91 ± 6.25, *b* = 0.36, *t* = 1.43, *p* = 0.155, 95% CI = [−3.40, 21.23]).

#### Consequences of Blame.

The blame from a blamer who demonstrated good moral commitment was more likely to be accepted by the blamee than the blame from a blamer who demonstrated bad moral commitment (*B* = 15.85 ± 5.53, *b* = 0.57, *t* = 2.87, *p* = 0.005, 95% CI = [4.95, 26.76]) (Figure 1C), and tended to elicit stronger self-conscious emotions (e.g., guilt, shame, embarrassment) in the blamee (*B* = 8.31 ± 4.17, *b* = 0.40, *t* = 1.99, *p* = 0.048, 95% CI = [0.09, 16.53]) (Figure 1E). Neither the main effect of type of rule, nor the interaction between type of rule and commitment was significant. Our manipulations had no significant effect on blamee’s willingness for behavioral improvement (Figure 1D).

### Discussion

In Study 1, we examined the effects of a blamer’s commitment to the same or a different moral rule as the one they blame the blamee for violating on the perception of the blamer’s moral standing to blame. We first showed that our measures of moral standing to blame had good internal reliability, which laid the foundation for the following studies and for future research on this topic. Not surprisingly, observers judged the blamer who committed similar wrongdoings as the blamee to have less standing to blame the blamee, even when the blamee was objectively blameworthy, supporting the philosophical conjecture that there is more to an act of blame than the blamee and the condemned behavior (Fritz & Miller, 2018; Roadevin, 2018; Todd, 2019; Tognazzini & Coates, 2024; Wallace, 2010); the moral commitment of the blamer also matters for important aspects of the act of blame such as the reception of the blame and its consequences. Interestingly, whether the blamer demonstrates bad commitment to the same or different rule than the one that they blame the blamee for violating does not matter too much for the perception of their moral standing to blame.

## STUDY 2

Study 1 initially explored the conceptual landscape of moral standing to blame and the moral consequences of a standingless blame. The key finding from Study 1 is that blamer’s good versus bad moral commitment has a strong effect on the perception of their moral standing to blame. In Study 2 and Study 3, we further investigated a nuanced question—what is observers’ default assumption about a blamer’s moral standing to blame, when the blamer has not had a chance to explicitly demonstrate their commitment to a given moral rule? Do blamers need to earn moral standing to blame by actively demonstrating good commitment to the moral rule, or do they just lose moral standing to blame by actively demonstrating bad commitment? Does the nature of the moral issue in question (e.g., straightforward vs. complicated) matter? With regard to the last question, moral knowledge and experience with a moral issue may be relevant. Individuals may share some universal moral knowledge (e.g., harm/care; cf. Graham et al., 2013), but specific moral knowledge could not be easily gained without relevant past experience (Levine & Moreland, 1994; Moreland & Levine, 1982; Pinto et al., 2010).

We manipulated whether the blamer has faced the same moral issue as the blamee. Specifically, in Study 2, the blamed behavior occurred in a certain relationship (e.g., between romantic partners or between a pet owner and their pet). We manipulated whether the blamer has the same social relationship in which the blamee’s blameworthy behaviors occurred. The blamer who does not have the said social relationship thus has not had the chance to actively demonstrate their commitment to the moral rule they blame the blamee for violating (No experience blamer hereafter). For the blamer who has the said social relationship, they either demonstrate good or bad commitment to the moral rule they blame the blamee for violating (Good blamer and Bad blamer hereafter). Based on the moral knowledge/experience hypothesis, we predicted that the No experience blamer would have some degree of moral standing between the Good blamer and the Bad blamer.

### Materials and Methods

#### Participants.

We recruited participants online using Prolific Academic. A priori power analysis indicated that we would need at least 69 participants per condition to detect a medium effect (f = 0.25) for the main effect of a one-way ANOVA with a power (1 − *β*) of 0.9 (*α* = 0.05). As we pre-registered (https://aspredicted.org/5SZ_RHY), two hundred twenty participants were recruited from Prolific, nine of whom were excluded due to failure in the attention check questions, leaving a final sample of 211 participants (115 or 54.5% females, *M*_*age*_ = 32.57, *SD*_*age*_ = 12.40).

#### Experimental Design and Materials.

This study adopted a 3-level between-subjects design. Two independent vignettes were created, each with three versions corresponding to the three experimental conditions (Good blamer, Bad blamer, and No experience blamer). This was to rule out the possibility that condition-induced effects were driven by any idiosyncratic features of any given vignette. Participants were randomly assigned to one of the six scenarios. The complete vignettes can be found in the Supplementary Materials.

Participants were asked to read a short vignette about two individuals. Within each vignette, the blamee explains that they committed a moral transgression for which the blamer blames them for. As an example, here are the full vignettes for each condition where the blamee is someone who has hurt their pet out of frustration. All the three versions (condition) start with the following background information:

**James** and **Mark** are coworkers at the mall. During one of their breaks, **Mark** tells **James** that he will sometimes hit his pet dog out of frustration when it doesn’t listen and becomes annoying.

Then the Good blamer condition continues:

**James**, who never hits his own dog when it misbehaves, blames **Mark** for what **Mark** has done.

The Bad blamer condition continues:

**James**, who sometimes hits his own dog when it misbehaves, blames **Mark** for what **Mark** has done.

Finally, the No experience blamer condition continues:

**James**, who doesn’t own a dog himself, blames **Mark** for what **Mark** has done.

### Results

#### Preliminary Analysis.

The blamee’s behavior was perceived to be quite blameworthy across all the three conditions (mean blameworthiness rating ranges between 84 and 90 on a slider scale of 0–100, where 0 = *not at all blameworthy*, 100 = *extremely blameworthy*). There was no significant difference in blameworthiness judgments across conditions (*F*(2, 208) = 1.25, *p* = 0.288). The blamee’s behavior was perceived to be very much a matter of morality (mean rating ranges between 79 and 82 on a scale of 0–100, where 100 = *very much a matter of morality*). Again, there was no significant difference across conditions (*F*(2, 207) = 0.190, *p* = 0.827).

#### Moral Standing to Blame.

We carried out the pre-registered linear regression analysis with the composite moral standing score as the dependent variable and blamer condition as the key independent variable (i.e., fixed-effect predictor). Vignette version was included as a random intercept. We believe that the content of the vignettes (e.g., someone cheating on their romantic partner vs. someone mistreating their pets) should not have a meaningful or systematic impact on the pattern of the results. In other words, the effects of the experimental manipulation (e.g., blamer’s commitment etc.), which was included as a fixed-effect predictor, should generalize across different idiosyncratic situations. By including vignette version as a random effect, we would be more confident that significant fixed effects are more generalizable.

**Figure 2.**
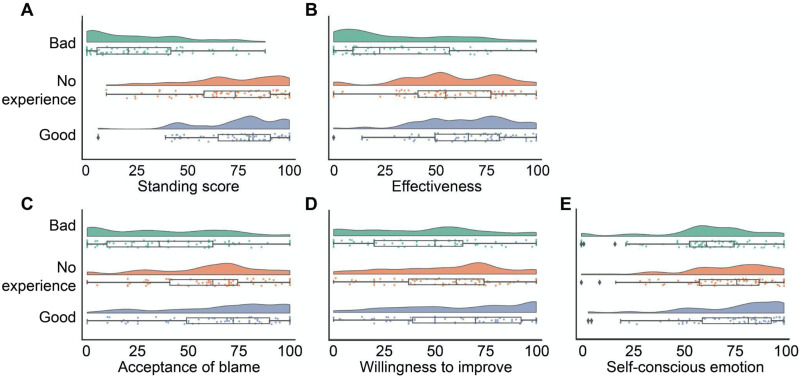
Results of Study 2. Primary dependent variables as function of blamer’s experience with the moral rule that they blame the blamee for.

We also included the blameworthiness of the blamee’s behavior and participants’ demographics (i.e., age, gender, socioeconomic status) as covariates. Excluding the demographic covariates did not change the pattern of results (Table S1). We found that the main effect of blamer condition was significant, such that the Bad blamer had the lowest perceived moral standing to blame (*B* = −43.61 ± 4.80, *b* = −1.38, *t* = −9.08, *p* < 0.001, 95% CI = [−53.14, −34.08] relative to the No experience blamer; *B* = −49.33 ± 4.90, *b* = −1.56, *t* = −10.06, *p* < 0.001, 95% CI = [−59.06, −39.60] relative to the Good blamer). Although, as we predicted, the No experience blamer was perceived to have lower moral standing to blame than the Good blamer, the difference was not statistically significant (*B* = −5.72 ± 5.38, *b* = −0.18, *t* = −1.06, *p* = 0.290, 95% CI = [−16.40, 4.96]; Figure 2A). This indicates that observers seem to assume that everyone has the moral standing to blame an obvious, uncontroversial blameworthy behavior, until the blamer actively demonstrates bad commitment to the moral rule.

#### Effectiveness and Consequences of Blame.

The effectiveness of blame, acceptance of the blame, willingness to improve, and self-conscious emotions elicited by the blame all showed a similar pattern as the standing to blame score (Figures 2B–E). While the blame from the Bad blamer was always perceived to be less effective, less accepted, elicited less willingness to improve, and less self-conscious emotions than the blame from the Good blamer, the No experience blamer was in between the Good blamer and Bad blamer conditions. Similar to the results of moral standing to blame, the Good blamer’s blame showed no significant difference compared with the No experience blamer’s blame on these dimensions either (see Table 1 for detailed statistics).

**Table 1.** Results of regression models regarding effectiveness and consequences of blame (Study 2)

Model	Coefficient *B* (*SE*)	95% CI of *B*	Standardized coefficient *b*	*t*-value
*Effectiveness*				
Bad vs. No experience	−21.44 (4.64)	[−30.60, −12.29]	−0.71	−4.61***
Bad vs. Good	−27.69 (4.70)	[−36.96, −18.42]	−0.91	−5.89***
No experience vs. Good	−6.26 (4.75)	[−15.63, 3.12]	−0.21	−1.32
*Acceptance*				
Bad vs. No experience	−21.09 (4.79)	[−30.53, −11.65]	−0.69	−4.41***
Bad vs. Good	−27.87 (4.84)	[−37.44, −18.32]	−0.91	−5.75***
No experience vs. Good	−6.79 (4.93)	[−16.51, 2.93]	−0.22	−1.38
*Improvement*				
Bad vs. No experience	−11.04 (4.79)	[−20.85, −1.22]	−0.36	−2.22*
Bad vs. Good	−19.32 (5.04)	[−29.26, −9.38]	−0.63	−3.83***
No experience vs. Good	−8.28 (5.12)	[−18.39, 1.82]	−0.27	−1.62
*Self-conscious emotion*				
Bad vs. No experience	−10.46 (4.14)	[−18.61, −2.30]	−0.42	−2.53*
Bad vs. Good	−15.33 (4.19)	[−23.58, −7.07]	−0.61	−3.66***
No experience vs. Good	−4.89 (4.25)	[−13.26, 3.52]	−0.19	−1.15

* *p* < 0.05.

*** *p* < 0.001.

### Discussion

Some philosophers argue that having faced the same moral dilemma or temptations, and indeed having committed a wrongdoing in those situations, provides the blamer with first-hand experiences of what is involved in those moral choices and what it means to err in those situations (Bell, 2013). Accordingly, these blamers should be more knowledgeable and competent when criticizing and blaming others for the same moral failure. In that sense, those who have faced the same moral issue may have privileged access to a specific area of moral knowledge, thereby having more moral standing to blame others’ failure in that domain. However, our findings did not support the conjecture that moral knowledge about a particular issue contributes significantly to the *perception* of moral standing (it is a separate issue whether a blamer’s moral knowledge *should* be a factor of the blamer’s moral standing in normative sense)—the Bad blamer was perceived to have the lowest moral standing to blame, even lower than the No experience blamer. It is possible that the moral issues we adopted in this study are straightforward enough that does not require any sophisticated moral knowledge or direct experience to understand and comment on. It is also possible that merely having the same moral experience and violating the same moral rule itself does not automatically confer additional moral standing to the blamer—how the blamer reacts to their past wrongdoing may also matter. We will address these possibilities in Study 3 through Study 5.

## STUDY 3

Study 2 demonstrates that even when a blamer has no direct experience with the moral issue in question and has not had a chance to demonstrate either good or bad commitment to the moral rule, they are still perceived to have the same moral standing to blame as someone who has demonstrated good commitment. This seems to suggest that moral knowledge and experience does not matter too much for a blamer’s moral standing to blame. To directly test the hypothesis in a stronger way, in Study 3 we adopted more complicated and controversial moral issues, where the judgment of right and wrong is not straightforward. Thus, it would be more challenging for someone who is privileged to not face such a moral issue to have moral knowledge or experience to fully understand the intentions, motives, and constraints that the blamee faces. We hypothesize that having the privilege of not facing the moral issues, and thus having no direct experience, will dampen the blamer’s standing to blame the blamee.

### Materials and Methods

#### Participants.

We recruited participants online using Prolific Academic. We pre-registered a sample of 70 American participants for each condition, consistent with Study 3 (https://aspredicted.org/VJ3_Q24). Two hundred and ten participants were recruited, among whom nine were excluded due to failure in the attention check questions, leaving a final sample of *N* = 201 (145 or 72.1% females, *M*_*age*_ = 33.6, *SD*_*age*_ = 12.5).

#### Experimental Design and Materials.

As Study 2, this study had a 3-level between-subjects design where we manipulated whether the blamer had the privilege of not facing the moral issues as the blamee does (“Privileged”), or faced the same moral issue as the blamee and demonstrated good (“Good”) or bad (“Bad”) commitment. Two unrelated vignettes were created, each with three versions corresponding to the three experimental conditions. Participants were randomly assigned to one of the six scenarios. The complete vignettes can be found in the Supplementary Materials.

The procedure was identical to Study 2. As an example, here are the full vignettes for each condition where the blamee is someone who is from a low-income family and steals from the supermarket where he works to support this family. All the three versions (condition) start with the following background information:

**James** and **Mark** are coworkers at a local supermarket. **Mark** is from a low-income family. He occasionally does not have enough food to eat. During one of their breaks, **Mark** tells **James** about his dire financial situation and that he sometimes steals from the supermarket they work at to support his family.

Then the successfully resisting (“Good”) condition continues:

**James**, who is also from a low-income family similar to **Mark**’s but has never stolen anything before, blames **Mark** for stealing from the supermarket.

The failure (“Bad”) condition continues:

**James**, who is also from a low-income family similar to **Mark**’s and has stolen from the supermarket too, blames **Mark** for stealing from the supermarket.

Finally, the no experience (“Privileged”) condition continues:

**James**, who is from a wealthy family and has never stolen anything before, blames **Mark** for stealing from the supermarket.

### Results

#### Manipulation Checks.

The blamee’s behavior was perceived to be moderately blameworthy across all the three conditions (mean blameworthiness rating ranges between 45 and 55 on a scale of 0–100, where 100 = *extremely blameworthy*). There was no significant difference in blameworthiness judgments across conditions (*F*(2, 197) = 2.90, *p* = 0.067). The blamee’s behavior was perceived to be somewhat a matter of morality (mean rating ranges between 42 and 55 on a scale of 0–100, where 100 = very much a matter of morality). Again, there was no significant difference across conditions (*F*(2, 197) = 2.78, *p* = 0.064).

#### Moral Standing to Blame.

**Figure 3.**
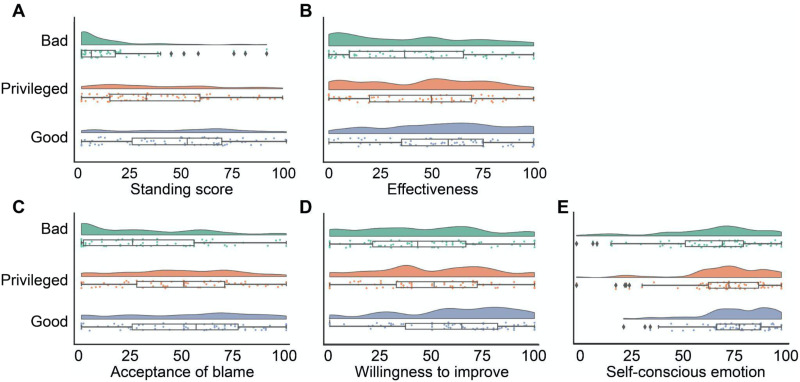
Results of Study 3. Primary dependent variables as function of blamer’s experience with the moral rule that they blame the blamee for.

As in Study 2, we carried out linear regression analysis with the composite moral standing score as the dependent variable, and blamer condition as the key independent variable. As pre-registered, we also included the moral evaluation of the blamee’s behavior and participants’ demographics (i.e., age, gender, socioeconomic status) as covariates. Excluding the demographic variables did not change the pattern of results (Table S1). We found that the main effect of blamer condition was significant (*F* (2, 194) = 39.57, *p* < 0.001), such that the Bad blamer had the lowest perceived moral standing to blame (*B* = −25.21 ± 3.64, *b* = −0.85, *t* = −6.92, *p* < 0.001, 95% CI = [−32.26, −18.16] relative to the Privileged blamer; *B* = −30.62 ± 3.69, *b* = −1.03, *t* = −8.29, *p* < 0.001, 95% CI = [−37.77, −23.48] relative to the Good blamer) (Figure 3A). The Good blamer was not perceived to have significantly more moral standing to blame than the Privileged blamer (*B* = 5.41 ± 3.68, *b* = 0.18, *t* = 1.47, *p* = 0.143, 95% CI = [−1.71, 12.53]).

Did the participants perceive the blamer who has not had a chance to demonstrate their commitment (i.e., the No experience blamer in Study 2 and the Privileged blamer in Study 3) to have more similar moral standing as the Bad blamer when the moral issues required more intricate and sophisticated moral knowledge (Study 3) than when the moral issues were more common sense (Study 2)? Indeed, in a supplemental study we recruited an independent group of participants (*N* = 299) to read the vignettes used in Study 2 and Study 3 and evaluated how much special knowledge would be needed for someone to appropriately blame the blamee in each vignette (for details, please see Supplemental Study in Supplementary Materials). We found that the participants judged that more special moral knowledge and direct experience would be needed to appropriately blame the blamees in the vignettes adopted in Study 3 than those in Study 2 (*B* = 29.38 ± 1.62, *b* = 1.17, *t* = 18.19, *p* < 0.001, 95% CI = [26.21, 32.55]), even after controlling for the blameworthiness of the blamee’s behaviors in the two studies (*B* = 16.13 ± 1.63, *b* = 0.64, *t* = 9.90, *p* < 0.001, 95% CI = [12.99, 19.40]). With this supportive evidence, we combined data from Study 2 and Study 3, and ran a regression model with three critical predictors: the main effect of condition (Good blamer vs. No experience/Privileged blamer), the main effect of study, and the interaction between these two factors. Participants’ demographic variables and blameworthiness judgment of the blamee were included as control variables. The interaction between condition and study was significant (*F*(1, 271) = 13.50, *p* < 0.001; *B* = 18.84 ± 5.13, *b* = 0.59, *t* = 3.67, *p* < 0.001, 95% CI = [8.74, 28.93]). Specifically, the moral standing to blame difference between the unknown commitment blamer condition (i.e., the No experience blamer in Study 2 and the Privileged blamer in Study 3) and the Bad blamer condition was smaller in Study 3 (*B* = 25.25 ± 3.69, 95% CI = [17.98, 32.51]) than in Study 2 (*B* = 44.08 ± 3.61, 95% CI = [36.98, 51.19]). This finding provides evidence for the role of moral knowledge and experience in the perception of a blamer’s moral standing to blame, especially when the moral issue in question is complicated.

#### Effectiveness and Consequences of Blame.

The effectiveness of blame, acceptance of the blame, willingness to improve, and self-conscious emotions elicited by the blame all showed a similar pattern as the standing to blame score (Figures 3B–E). While the blame from the Bad blamer was perceived to be less effective, less accepted, elicited less willingness to improve less least self-conscious emotions than the blame from the Good blamer, there was no significant difference in these regards between the Bad and the Privileged blamer, except for willingness to accept the blame, in which case the Bad blamer was significantly lower than the Privileged blamer (see Table 2 for statistics). One possibility for the uniqueness of the acceptance measure is that the other DVs (willingness to improve, self-conscious emotions) are more about how the blamee reflects on their own moral wrongdoing, and are more detached from their relationship with the blamer. Therefore, the moral commitment of the blamer may not matter a lot. In contrast, acceptance of blame and moral standing emphasize the role of the blamer (e.g., acceptance of blame from this blamer). Future work is needed to empirically test this speculation.

**Table 2.** Results of regression models regarding effectiveness and consequences of blame (Study 3)

Model	Coefficient *B* (*SE*)	95% CI of *B*	Standardized coefficient *b*	*t*-value
*Effectiveness*				
Bad vs. Privileged	−5.27 (4.67)	[−3.78, 14.31]	−0.17	−1.13
Bad vs. Good	−12.29 (4.74)	[−21.45, −3.12]	−0.40	−2.59*
Privileged vs. Good	−7.02 (4.72)	[−16.14, 2.11]	−0.23	−1.49
*Acceptance*				
Bad vs. Privileged	−16.76 (4.54)	[−25.56, −7.97]	−0.55	−3.69***
Bad vs. Good	−16.71 (4.61)	[−25.62, −7.80]	−0.54	−3.63***
Privileged vs. Good	0.06 (4.59)	[−8.82, 8.93]	0.00	0.01
*Improvement*				
Bad vs. Privileged	−3.75 (4.73)	[−12.90, 5.40]	−0.13	−0.79
Bad vs. Good	−10.37 (4.79)	[−19.64, −1.11]	−0.35	−2.17*
Privileged vs. Good	−6.63 (4.77)	[−15.86, 2.61]	−0.23	−1.39
*Self-conscious emotion*				
Bad vs. Privileged	−6.46 (3.51)	[−13.25, 0.33]	−0.30	−1.84
Bad vs. Good	−8.91 (3.56)	[−15.78, −2.03]	−0.41	−2.51*
Privileged vs. Good	−2.44 (3.54)	[−9.30, 4.41]	−0.11	−1.15

* *p* < 0.05.

*** *p* < 0.001.

Of note, when comparing across Study 2 and Study 3, we found that the effectiveness of the unknown commitment blamer was more similar to the Bad blamer in Study 3 than in Study 2, as indicated by a significant study-by-condition interaction (*F*(1, 271) = 7.51, *p* = 0.007; *B* = 17.87 ± 6.52, *b* = 0.59, *t* = 2.74, *p* = 0.007, 95% CI = [5.03, 30.72]). Specifically, the effectiveness of blame difference between the unknown commitment blamer condition and the Bad blamer condition was smaller in Study 3 (*B* = 5.28 ± 4.70, 95% CI = [−3.96, 14.53]) than in Study 2 (*B* = 23.16 ± 4.59, 95% CI = [14.12, 32.20]).

### Discussion

The significant difference in perceived moral standing to blame between the Good and Bad blamer conditions once again supports the notion that good moral commitment is crucial for blamer’s moral standing to blame. Interestingly, even the Privileged blamer’s moral standing to blame was not significantly lower than the Good blamer, who demonstrated good moral commitment, and was significantly higher than the Bad blamer, who demonstrated bad moral commitment. This again supports the notion that observers assume that the blamer has moral standing to blame unless the blamer actively demonstrates their lack of moral commitment through their explicit rule violation behaviors. For those who have not had the chance to demonstrate the lack of moral standing, the observers tend to give them “the benefit of the doubt”.

## STUDY 4

In Study 4 we further investigated the observers’ default assumption of a blamer’s moral standing to blame when the blamer has not had a chance to demonstrate good or bad commitment to a moral rule. In this study, we considered a situation different from the one that has already been studied in Study 2 and Study 3, namely, identity-based moral rule. Some moral rules only regulate behaviors of individuals who assume certain identity (e.g., not eating certain foods for some religious believers and vegans). Some behaviors are not seen as morally bad if performed by someone who does not share a certain identity. For example, eating meat does not demonstrate bad commitment to veganism for someone who does not consider themselves as a vegan, as it would do for someone who claims to be a vegan. This investigation would also give us an opportunity to compare identity-based standing (i.e., psychological standing; Effron & Miller, 2015; Miller & Effron, 2010) with standing based on the blamer’s moral beliefs and commitment. Would someone who shares a certain identity (thereby subject to the identityspecific moral rule) but demonstrates bad commitment to the identity-specific moral rule have higher or lower moral standing to blame than someone who does not share that identity?

Study 4 would also replicate and clarify some of the conclusions from Study 2 and Study 3. There were several limitations in Study 2 and Study 3. The behaviors of the No experience/Privileged blamer and the Bad blamer were not matched. By design, their behaviors could not be matched because the blamer in the No experience/Privileged condition has not faced the same moral situations as the blamee. Therefore, it is unclear whether the differences in the perceived moral standing to blame and the consequences of the blame were due to differences in the blamers’ behaviors or due to difference in the relation between the blamer and the moral rule. In Study 4, we aimed to address this confounding issue and conceptual ambiguity. Specifically, we kept the behaviors of the blamee and the blamer in the same domain across conditions (i.e., eating meat or practicing veganism), and varied the moral rules that the blamer refers to in their blame (vegan-specific vs. generic). Another advantage of this design is that performing certain behaviors (e.g., eating meat) does not necessarily reflect badly on the blamer’s moral character. This allows us to dissociate identity-based standing and moral standing to blame.

We hypothesized that: 1) a blamer who does not claim to have an identity (e.g., being a vegan) will have more moral standing to blame a blamee for violating an identity-specific moral rule (e.g., not eating meat for vegan) than a blamer who claims to have that identity and demonstrates bad commitment to the identity-specific moral rule; 2) a blamer’s same behavior (e.g., eating meat) may be considered as not demonstrating either good or bad commitment to some rules (i.e., an identity-specific rule, such as veganism) if the blamer does not have the relevant identity, but at the same time may be considered as demonstrating bad commitment to other rules (e.g., a generic rule, such as obligation for environmental protection) even when the blamer does not have any specific identity. The blamer’s perceived moral standing to blame would vary according to the rule they cite to blame the blamee’s behavior.

### Materials and Methods

#### Participants.

A prior power analysis indicated that to detect a medium-to-large effect (*d* = 0.6) for the comparison of moral standing between the two blame types (i.e., identity-specific blame vs. generic blame) in the non-member conditions, we needed at least 60 participants per condition to detect this effect with a power (1 − *β*) of 0.9. Four hundred and six American participants were recruited from Prolific (https://aspredicted.org/LPL_XLF), 43 of whom were excluded due to failing the attention check questions, leaving a final sample of 363 (192 or 52.9% females, *M*_*age*_ = 34.5, *SD*_*age*_ = 12.6).

#### Experimental Design and Materials.

We adopted a morally relevant identity, veganism, as the basis of the identity-specific moral violation. Not consuming animal products is a moral rule that applies to people who identify as vegan (Caviola et al., 2019; Rosenfeld et al., 2020), but not to people who do not identify as vegan. In all the scenarios, the blamee is a self-identified vegan who eats meat (i.e., a bad vegan). The first factor we manipulated in this study was the type of blamer, which had three levels: Good Vegan blamer, who identifies as vegan and does not eat meat; Bad Vegan blamer, who identifies as vegan but consumes meat; and Non-Vegan blamer, who does not identify as vegan and eats meat. Orthogonal to the membership status, we manipulated the type of blame, or the moral rule the blamer cites to blame the blamee. In the specific blame conditions, the blamer blames the blamee for violating a moral rule that is specific to the blamee’s identity (e.g., “It is wrong for a vegan to eat meat.”). In the generic blame conditions, the blamer cites a moral rule to blame the blamee, which is not specific to the blamee’s identity (e.g., “Eating meat harms the environment.”). Therefore, the study had a 2 (blame type: Specific vs. Generic) by 3 (blamer type: Good vegan, Bad vegan, Non-vegan) factorial between-subject design. The complete vignettes can be found in the Supplementary Materials. Each participant was presented with one of the six versions (i.e., conditions) of the vignette and were then asked to complete our set of measures, as described in detail in Study 1.

### Results

#### Manipulation Checks and Preliminary Analysis.

To make sure our manipulations of blame type and blamer type were understood as we expected, we carried out the following manipulation check analyses. First, we asked the participants to indicate the extent to which the blamer should be expected to practice veganism. We found that the Good vegan blamer (*B* = 40.63 ± 5.24, *b* = 1.06, *t* = 7.75, *p* < 0.001, 95% CI = [30.32, 50.94]) and Bad vegan blamer (*B* = 33.01 ± 5.40, *b* = 0.86, *t* = 3.61, *p* < 0.001, 95% CI = [22.38, 43.63]) were more strongly expected to practice veganism than the Non-vegan blamer. Second, the Non-vegan blamer should be more strongly expected to conform to the moral rule underlying the generic blame (i.e., environmental protection) than the specific blame (i.e., a veganism rule). This is exactly what we observed (*F*(1, 129) = 4.22, *p* = 0.042).

The blamee’s behavior was perceived to be mildly blameworthy across all the six conditions (mean blameworthiness rating ranges between 27 and 45 on a slider scale where 0 = *not blameworthy at all*, 100 = *extremely blameworthy*). There was no significant difference in blameworthiness judgments across conditions (*F*(1, 361) = 1.65, *p* = 0.200). The blamee was not perceived to be a good vegan (mean rating ranges between 27 and 33 on a slider scale of 0–100, where 0 = *not a good vegan* at all, 100 = *an extremely good vegan*). Again, there was no significant difference in the perception of how good a vegan the blamee is across conditions (*F*(1, 361) = 0.540, *p* = 0.463). Moreover, the average rating of this item was significantly below the midpoint of the scale (i.e., 50 = *somewhat a good vegan*) for all the six conditions (*t*s < −5.80, *p*s < 0.001).

#### Moral Standing to Blame.

To examine the effects of type of blame (identity-specific vs. generic), type of blamer (Good Vegan, Bad Vegan, Non-Vegan), and their interaction on the perception of blamer’s moral standing to blame, we estimated a linear model with the two factors and their interaction as the key independent variables. As pre-registered, we also include the judgment of the blamee’s blameworthiness, the participants’ demographic variables, (i.e., age, gender, and socioeconomic status) as covariates. The pattern of results remained when excluding these demographic covariates in the regression (Table S1). The main effect of blame type (identity-specific vs. generic) was significant (*F* = 34.56, *p* < 0.001), such that blamers who cited the identity-specific rule to blame were perceived to have more moral standing to blame than the blamers who cited the generic rule to blame (*B* = 16.00 ± 4.43, *b* = 0.47, *t* = 3.61, *p* < 0.001, 95% CI = [7.29, 24.71]). Blamer type also had a significant main effect on moral standing to blame (*F* = 34.56, *p* < 0.001), such that the Good Vegan blamer has more moral standing to blame than both the Bad Vegan blamer (*B* = 28.29 ± 4.28, *b* = 1.00, *t* = 6.60, *p* < 0.001, 95% CI = [19.86, 36.72]) and the Non-Vegan blamer (*B* = 25.28 ± 3.91, *b* = 0.89, *t* = 6.47, *p* < 0.001, 95% CI = [17.60, 32.96]). Critically, blame type and blamer type had a significant interactive effect on the perception of moral standing to blame (*F* = 4.39, *p* = 0.013).

To unpack this interaction pattern, we carried out two separate linear regression models to examine the effect of blamer type on the perception of moral standing to blame separately for each type of blame. We predicted that, when the blamer does not share the relevant identity (i.e., being a vegan), they should have some degree of moral standing to blame, even when their behavior is the same as the Bad Vegan. That is because the Non-Vegan’s behavior does not demonstrate their bad commitment to the rule (the rule does not apply to them in the first place) as the Bad Vegan’s behavior does. First, as a manipulation check (“To what extent is [blamer’s name] morally required to do well regarding the reason he cites to blame [blamee’s name]?”, 0 = *Not at all required*, 100 = *Very much required*), we confirmed that the participants believed that the identity-specific moral rule is less applicable to the Non-Vegan blamer than the generic moral rule (*B* = −13.00 ± 6.24, *b* = −0.36, *t* = −2.09, *p* = 0.039, 95% CI = [−25.34, −0.66]). We then demonstrated that in the identity-specific blame conditions, the perceived moral standing to blame of the Non-Vegan blamer was significantly lower than the Good Vegan blamer (*B* = 10.44 ± 5.04, *b* = −0.36, *t* = 2.07, *p* = 0.040, 95% CI = [−20.39, −0.49]) and significantly higher than the Bad Vegan blamer (*B* = 13.10 ± 5.03, *b* = 0.45, *t* = 2.60, *p* = 0.010, 95% CI = [3.17, 23.02]). This suggests that when observers evaluate a blamer’s moral standing, the blamer’s demonstrated commitment seems to be more important than the blamer’s identity.

**Figure 4.**
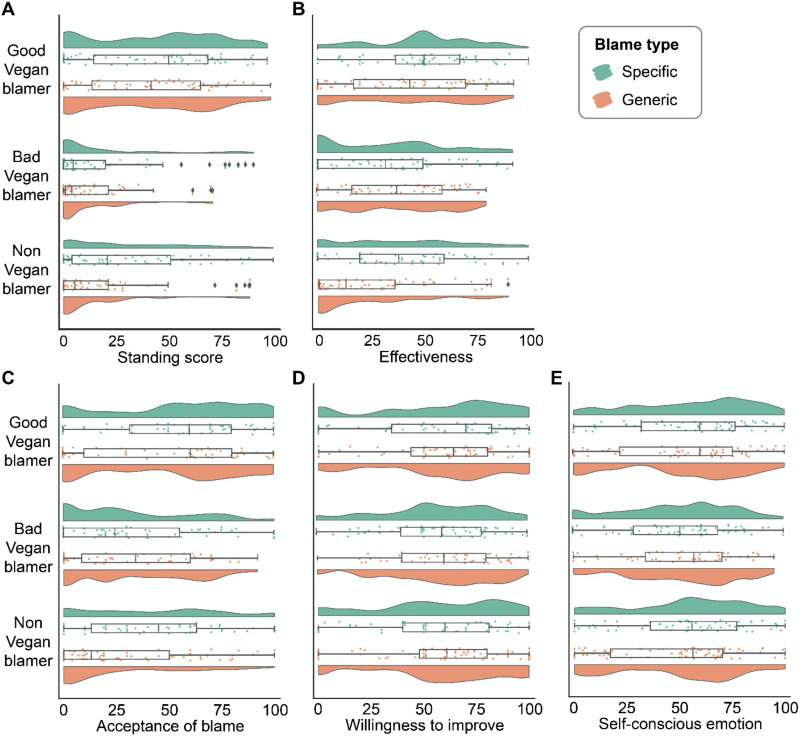
Results of Study 4. Primary dependent variables as a function of type of blame and type of blamer.

When the Non-Vegan blamer cites a rule that is not specific to the vegan identity (i.e., in the generic blame conditions), in which case the Non-Vegan blamer’s behavior demonstrates their bad commitment to the rule (i.e., eating meat demonstrates bad commitment to the moral rule “one should not harm the environment” regardless of whether one identifies as vegan or not), there was no difference in the perceived moral standing to blame between the Non-Vegan blamer and the Bad Vegan blamer (*B* = −1.58 ± 4.31, *b* = −0.06, *t* = −0.37, *p* = 0.715, 95% CI = [−10.09, 6.94]). The Good Vegan blamer was perceived to have higher moral standing to blame than both the Non-Vegan blamer (*B* = 26.55 ± 3.95, *b* = 0.97, *t* = 6.72, *p* < 0.001, 95% CI = [18.75, 34.35]) and the Bad Vegan blamer (*B* = 28.77 ± 4.35, *b* = 1.05, *t* = 6.62, *p* < 0.001, 95% CI = [20.19, 37.35]). Looking at the interaction from a different angle, blame type had a significant effect only on the Non-Vegan blamer’s moral standing to blame (Specific > Generic: *B* = 13.35 ± 3.81, *b* = 0.47, *t* = 3.51, *p* < 0.001, 95% CI = [5.86, 20.84]), but not on the Good Vegan or the Bad Vegan blamer’s moral standing to blame (Figure 4A). Note that the Non-Vegan blamer’s behavior (i.e., eating meat) is the same in the two conditions, therefore the difference in moral standing to blame cannot be attributed to the difference in Non-Vegan blamer’s behavior.

#### Effectiveness of Blame.

Blamer type had a significant main effect on the perceived effectiveness of blame (*F* = 8.69, *p* < 0.001), such that the Non-Vegan blamer’s blame was perceived as less effective than both the Good Vegan blamer (*B* = −22.82 ± 4.66, *b* = −0.79, *t* = −4.89, *p* < 0.001, 95% CI = [−31.99, −13.64]) and the Bad Vegan blamer (*B* = −15.35 ± 4.79, *b* = −0.53, *t* = −3.21, *p* = 0.001, 95% CI = [−24.76, −5.93]). Interestingly, the interaction between blamer type and blame type was significant (*F* = 6.17, *p* = 0.002), such that the Non-Vegan blamer’s blame was less effective than that of the Good Vegan blamer and the Bad Vegan blamer only when they cited generic rule to blame (*B* = −23.65 ± 4.48, *b* = −0.85, *t* = −5.28, *p* < 0.001, 95% CI = [−32.50, −14.81] for the comparison between the Non-Vegan blamer and the Good Vegan blamer; *B* = −16.15 ± 4.59, *b* = −0.58, *t* = −3.52, *p* < 0.001, 95% CI = [−25.22, −7.08] for the comparison between the Non-Vegan blamer and the Bad Vegan blamer), but not when they cite identity-specific rule to blame (*B* = −3.89 ± 4.98, *b* = −0.13, *t* = −0.78, *p* = 0.436, 95% CI = [−13.72, 5.95] for the comparison between the Non-Vegan blamer and the Good Vegan blamer; *B* = 4.43 ± 4.95, *b* = 0.15, *t* = 0.90, *p* = 0.372, 95% CI = [−5.34, 14.19] for the comparison between non-member and bad member) (Figure 4B). The main effect of blame type was not significant (*F* = 0.87, *p* = 0.351).

#### Consequences of Blame.

The main effect of blamer type on the blamee’s willingness to accept the blame was significant (*F* = 18.70, *p* < 0.001), such that the Good Vegan blamer’s blame was the most likely to be accepted by the blamee (*B* = 24.74 ± 4.83, *b* = 0.76, *t* = 5.12, *p* < 0.001, 95% CI = [15.23, 34.24] for the comparison between the Good Vegan blamer and the Non-Vegan blamer; *B* = 18.04 ± 5.30, *b* = 0.55, *t* = 3.40, *p* < 0.001, 95% CI = [7.62, 28.47] for the comparison between the Good Vegan blamer and the Bad Vegan blamer) (Figure 4C). The interaction between blamer type and blame type was marginally significant (*F* = 3.01, *p* = 0.050). To unpack the interaction pattern, we examined the effect of blame type for each type of blamer separately. The type of blame did not make a difference in blamee’s willingness to accept the Good Vegan’s blamer and Bad Vegan’s blame. This is conceivable because the moral rule applies to the Good Vegan (*B* = 0.02 ± 5.10, *b* = 0.00, *t* = 0.00, *p* = 0.997, 95% CI = [−10.01, 10.05]) and the Bad Vegan (*B* = −2.43 ± 5.20, *b* = −0.07, *t* = −0.47, *p* = 0.641, 95% CI = [−12.66, 7.81]) in both cases (i.e., identity-specific and generic). However, the type of blame did make a difference in blamee’s willingness to accept the Non-Vegan blamer’s blame. Non-Vegan blamer’s blame was more likely to be accepted when it was based on an identity-specific moral rule than generic moral rule (*B* = 13.33 ± 4.71, *b* = 0.41, *t* = 2.83, *p* = 0.005, 95% CI = [4.06, 22.59]). Our experimental manipulations had no effect on blamee’s willingness to improve or self-conscious emotion elicited by their moral wrongdoing (Figures 4D, 4E).

### Discussion

In Study 4, we further dissociated the effects of a given behavior and its moral implications on the perception of a blamer’s moral standing to blame. Specifically, a behavior (e.g., eating meat) does not in itself dampen a blamer’s moral standing to blame; this occurs only when the behavior clearly demonstrates the blamer’s bad commitment to the rule that they cite to blame the blamee for violating. We also demonstrated that the same behavior has different moral implications to the blamer’s commitment under different descriptions. Under a narrower, identity-specific description, eating meat violates the moral obligation dictated by the veganism identity. In contrast, under a broader, generic description, eating meat is bad for the environment, which should, at least in theory, apply to everyone. Therefore, under the identity-specific description, eating meat would only demonstrate bad commitment to vegan-specific rule for those who identify as vegan and accept the moral rule of not eating meat, but not to others; but it would be a moral failure even for non-vegan under the generic description. Such flexibility in interpreting the moral status of blamer’s behavior was reflected in the observers’ perceptions of the blamer’s moral standing to blame, suggesting that when evaluating a blamer’s moral standing to blame, observers make inference about the blamer’s commitment to the rule from the blamer’s behavior. The observers’ evaluation of the blamer’s moral standing to blame is relied on the inferred commitment more than on the behavior itself. These results thus provide stronger evidence for the moral commitment hypothesis.

These results have important implications for moral theories of moral standing to blame. Some philosophical work on moral standing to blame argues that uncommitted blamer (i.e., a blamer who does not accept a certain moral rule as applicable to themselves) and blamer who demonstrates bad commitment to a rule are equally standingless (Lippert-Rasmussen, 2023). Here, we provided quantitative and experimentally controlled comparison between uncommitted blamer and blamer who actively demonstrates bad commitment to the moral rule. Our result suggests that from a layperson’s perspective, an uncommitted blamer has more moral standing to blame someone who violates a moral rule than a blamer who demonstrates bad commitment to that rule.

## STUDY 5

The first four studies have investigated moral commitment as an important antecedent of the perception of moral standing to blame, and explored the influence of moral knowledge/experience on the perception of moral standing to blame. In particular, we asked what moral attributes of a blamer reduce observers’ perception of their moral standing to blame. The empirical findings thus far have lent strong and consistent support to the moral commitment hypothesis, namely, a blamer will be perceived to have low moral standing to blame if they demonstrate bad moral commitment to the moral rule that they blame the blamee for violating. In Study 5, we tested the moral commitment hypothesis from a different angle. Specifically, we examined whether and how addressing their demonstrated bad moral commitment (e.g., attitudes and emotional responses) influences the perception of the blamer’s moral standing to blame the same wrongdoing of others. Acknowledging one’s own wrongdoing and feeling/expressing guilt and regret have been proposed as an effective and morally legitimate way of addressing one’s past wrongdoing, and a signal of commitment to the moral rule (Lippert-Rasmussen, 2023; O’Connor et al., 2020; Todd, 2019). We therefore hypothesized that acknowledging one’s own wrongdoing and expressing guilt about it should help a morally problematic blamer to regain standing to blame.

### Materials and Methods

#### Participants.

A power analysis indicated that we would need at least 53 participants per condition to detect a medium effect (f = 0.25) for the main effect of an ANOVA with a power (1 − *β*) of 0.9 (*α* = 0.05). As we pre-registered (https://aspredicted.org/7QL_319), we recruited 339 participants from Prolific, eleven of whom were excluded due to failure in the attention check questions, leaving a final sample of 328 participants (166 or 50.6% females, *M*_*age*_ = 34.41, *SD*_*age*_ = 11.47).

#### Experimental Design and Materials.

Similar to the previous studies, participants were presented with vignettes and were asked to evaluate the target characters in the vignettes. We manipulated two factors in this study: whether the blamer chooses to admit that they committed the same moral wrongdoing themselves and whether the blamer feels guilty for committing the moral wrongdoing. This study also had a control condition in which participants read about a blamer who does not commit the same wrongdoing and thus has no need to admit or express guilt for a similar wrongdoing. Thus, this study had a 2 × 2 between-subjects design with an additional control condition.

Participants were asked to read a short vignette about two individuals. Within each vignette, participants read about two target characters: Amanda and Becky. For each condition besides the control condition, participants read about Amanda, who occasionally downloads music illegally, catching her roommate, Becky, downloading music illegally. In response, Amanda condemns and blames Becky for illegally downloading music. To show what participants read, in all conditions besides the control condition, the vignette began as follows:

**Amanda** and **Becky** are roommates. Loving music but not being able to purchase every song she likes; **Amanda** occasionally downloads music illegally from the Internet. One day, **Amanda** sees Becky downloading music illegally from the Internet. **Amanda** tells **Becky** that downloading music illegally from the Internet is wrong and blames **Becky** for doing it.

In the four main conditions, participants additionally read more about Amanda’s response. Specifically, participants were shown additional text that explained whether or not Amanda lied about downloading music illegally and whether or not Amanda felt guilty for doing so. Thus, in the Disclose and Guilty condition, the vignette continued,

**Amanda** admits that she downloads music illegally herself. In her heart, Amanda does feel really guilty about downloading music illegally.

In the Disclose and No guilt condition, the vignette instead continued,

**Amanda** admits that she downloads music illegally herself. In her heart, Amanda does not feel guilty about downloading music illegally.

In the Hidden and Guilty condition, the vignette instead continued,

Although **Amanda** downloads music illegally herself, she does not mention it to **Becky**. In her heart, **Amanda** does feel really guilty about downloading music illegally.

In the Hidden and No guilt condition, the vignette instead continued,

Although **Amanda** downloads music illegally herself, she does not mention it to **Becky**. In her heart, **Amanda** does not feel guilty about downloading music illegally.

Within the control condition, participants instead read the following vignette:

**Amanda** and **Becky** are roommates. Loving music but not being able to purchase every song she likes; **Amanda** sometimes has to choose very carefully which songs to purchase. One day, **Amanda** sees **Becky** downloading music illegally from the Internet. **Amanda** tells **Becky** that downloading music illegally from the Internet is wrong and blames **Becky** for doing it.

### Results

#### Manipulation Checks.

The blameworthiness judgments of the blamee’s behavior were within the “somewhat blameworthy” range across all the five conditions. The difference between the midpoint of the scale (i.e., 50 = somewhat blameworthy) and the average blameworthiness rating of neither condition was significant (∣*t*s∣ < 1.96, *p*s > 0.055). One-way ANOVA showed that the condition did not have a significant effect of blameworthiness judgments of the blamee’s behavior (*F*(4, 270) = 2.26, *p* = 0.063). No pairwise comparison was significant after Bonferroni correction of multiple comparisons.

Does acknowledging one’s past wrongdoing and feeling guilty about it make the blamer a morally better person in the eyes of observers? We asked the participants to evaluate the moral character of the blamer they read about (“How much do you think [blamer’s name] can be described by the following traits?” The three moral traits were “moral”, “kind”, and “trustworthy”. We averaged them to obtain a composite score; Yu et al., 2021). We ran a regression model with the main effects of disclosure, emotion type, and their interaction as the primary predictors. Participants’ demographic variables and blameworthiness judgment of blamee’s behavior were included as control variables. The main effect of disclosure was significant (*B* = 11.45 ± 3.22, *b* = 0.65, *t* = 3.56, *p* < 0.001, 95% CI = [5.11, 17.79]), such that the blamer who admitted their past wrongdoing was perceived to be a morally better person. The interaction effect was also significant (*B* = 10.17 ± 4.53, *b* = 0.58, *t* = 2.25, *p* = 0.026, 95% CI = [1.24, 19.11]), such that feeling guilty for their past wrongdoing made the blamer a morally better person only when the blamer admitted their past wrongdoing (*B* = 12.90 ± 3.22, *b* = 0.73, *t* = 4.01, *p* < 0.001, 95% CI = [6.56, 19.25]), but not when the blamer concealed their past wrong doing (*B* = 2.73 ± 3.21, *b* = 0.15, *t* = 0.85, *p* = 0.396, 95% CI = [−3.60, 9.06]). These results suggest that admitting one’s past wrongdoing and feeling guilty about it together improves the moral impression of blamer in the eyes of observers (i.e., better rule commitment).

#### Moral Standing to Blame.

**Figure 5.**
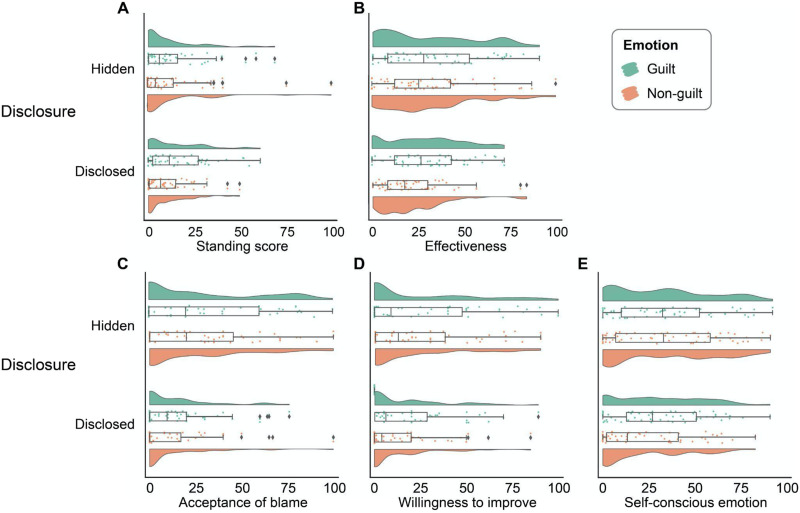
Results of Study 5. Primary dependent variables as function of whether blamer acknowledge their past wrongdoing and the emotion associated with the blame.

Not surprisingly, the blamer in the Control condition (i.e., no bad commitment) was perceived to have the highest standing to blame (53.89 ± 3.7; all comparisons with the other four conditions were significant after Bonferroni correction, *p*s < 0.001). For the four experimental conditions, we examined the effects of disclosure, emotion type, and their interaction on the moral standing to blame. Neither the main effect of disclosure nor the main effect of emotion was significant. However, partially supporting our prediction, the interaction between disclosure and emotion was marginally significant in the direction as we pre-registered (*B* = −7.44 ± 4.00, *b* = −0.46, *t* = −1.86, *p* = 0.064, 95% CI = [−15.33, 0.45]; Figure 5A). Specifically, when the blamer disclosed their past wrongdoing, feeling guilty gave them more standing to blame relative to feeling no guilt (*B* = 6.91 ± 2.84, *b* = 0.43, *t* = 2.43, *p* = 0.016, 95% CI = [1.30, 12.51]). When the blamer did not disclose their past wrongdoing, merely feeling guilty had no effect on their moral standing to blame (*B* = 0.53 ± 2.83, *b* = 0.03, *t* = 0.189, *p* = 0.851, 95% CI = [−5.05, 6.12]). This pattern mirrors that of the moral impression of the blamer, suggesting a potential link between improvement on rule commitment and increase in the perception of moral standing to blame. Excluding demographic covariates did not change the patterns of the results (Table S1).

One might argue that merely feeling guilty privately may not be a sufficient way to address one’s past wrongdoing and therefore may not boost the blamer’s standing to blame by a lot. To test the possibility that sincerely and openly expressing guilt may further elevate a blamer’s standing to blame over and above disclosure and feeling guilty privately, we recruited another group of participants to complete a new condition, where the blamer not only disclosed their wrongdoing, but also openly expressed their guilty feelings to the blamee (*N* = 53, 27 females, *M*_*age*_ = 36.6, *SD*_*age*_ = 11.1). We ran a linear regression model that only included the three disclosure conditions (i.e., non-guilt, feeling guilt, expressing guilt). We found that, similar to the feeling guilt condition, the expressing guilt condition had significantly higher standing to blame than the nonguilt condition (*B* = 5.85 ± 2.88, *b* = 0.39, *t* = 2.03, *p* = 0.044, 95% CI = [0.16, 11.53]), but there was no significant difference between the expressing and feeling guilt conditions (*B* = 1.51 ± 2.81, *b* = 0.10, *t* = 0.54, *p* = 0.591, 95% CI = [−4.04, 7.06]).

#### Effectiveness and Consequences of Blame.

The interaction between disclosure and emotion was not significant for perceived effectiveness of blame, blamees’ acceptance of blame, willingness to improve in the future, and self-conscious emotion as a consequence of the blame (Figures 5B–E). Blames are more likely to be accepted (*B* = 14.36 ± 4.68, *b* = 0.40, *t* = 3.07, *p* = 0.002, 95% CI = [5.13, 23.58]) when the blamer does not disclose their past wrongdoing. This is not surprising because from the blamee’s perspective, if the blamer does not disclose their past wrongdoing, there would be no way for the blamee to find out about the blamer’s bad moral commitment. The blamer would therefore be treated in the same way as a morally clearn blamer in the Control condition.

### Discussion

Replicating a consistent finding from the first four studies, here we found that blamers who have demonstrated bad moral commitment to the moral rule that they blame the blamee for violating were perceived to have lower standing to blame. More importantly, we showed that acknowledging one’s past wrongdoing and feeling/expressing guilt for it indeed helps a blamer to restore some standing to blame. We note that the effect of feeling and expressing guilt on the perception of moral standing to blame was small in this study. This may be an idiosyncratic issue with the vignette we used, such as the severity of the blamee’s and the blamer’s wrongdoing, the sincerity of guilt feeling and expression, and whether compensatory actions are taken in addition to guilt feeling and expression. Future research using a more diverse set of moral vignettes is needed to better address these questions.

## GENERAL DISCUSSION

What makes a blamer lack moral standing to blame? Across five studies, we found converging evidence for a “moral commitment” hypothesis—when the blamer demonstrates bad commitment to the moral rule they blame the blamee for violating, they are perceived to have low moral standing to blame. This finding provides evidence for the strong and consistent effect of commitment to the moral rule on a blamers’ moral standing to blame. Importantly, observers seem to assume that a blamer has moral standing to blame by default, unless the blamer actively demonstrates bad moral commitment to the rule that they blame the blamee for.

Our research has important theoretical and practical implications. From a theoretical perspective, our research complements the psychology literature on blame by emphasizing the importance of blamer-related (as opposed to blamee-related; Malle et al., 2014) factors in an act of blame. Our research also complements the work on psychological standing by showing that moral characteristics, in addition to identity-based characteristics (Miller & Effron, 2010), also matters when evaluating the legitimacy of a social act (e.g., blame, protest, intervention, etc.).

From a practical/applied perspective, our research has at least two implications. First, given the demonstrated importance of moral standing for how effective and acceptable a blame could be, a blamer needs to be mindful of their moral standing before blaming others. If their moral standing is compromised by their lack of commitment to a moral rule, they need to restore their moral standing before their blame can be taken seriously by the blamees. Second, moral standing could also be abused by blamees to fend off blame of their wrongdoing that they should take seriously. This could be particularly problematic in intergroup and international contexts, where it is uncommon for any collective blamer to have a clean moral record in history (see below for more discussion).

When directly comparing identity-based (psychological) standing and moral standing to blame (Study 1 and Study 4), we found that lack of moral standing had a stronger effect than lack of psychological standing on participants’ judgments. For example, not having the same identity as the blamee (i.e., lack of psychological standing) has less impact on a blamer’s moral standing to blame than having the same identity as the blamee but demonstrating bad commitment to the identity-specific moral rule. These findings, complementing the research on psychological standing (e.g., Effron & Miller, 2015; Miller & Effron, 2010), highlight moral standing as a distinct and important concept in the psychology of blame. Our goal here is to broaden the scope of empirical research on the psychological experience and sources of standing (i.e., feeling of legitimacy and entitlement). As we argued in the Introduction, psychological standing and moral standing are two conceptually overlapping but distinct constructs. More specifically, we aimed to identify critical moral characteristics that ordinary individuals consider when determining a blamer’s moral standing to blame and to evaluate the impact of a blamer’s lack of standing on their blame. Ascertaining the mechanisms through which social identity grants psychological standing in some situations (e.g., criticizing a policy of one’s own country) and moral characteristics grant moral standing in other situations (e.g., blaming someone for a wrongdoing that one has committed in the past) is an important empirical question that worth future research.

In addition, our studies also reveal nuanced effects of relevant moral knowledge and experience on the perception of a blamer’s moral standing to blame. Study 1 shows that as long as the blamer’s and the blamee’s wrongdoings are comparable in degree, the blamer’s bad commitment to the moral rule has comparable negative effect on the perception of the blamer’s moral standing to blame. A blamer’s commitment to a different moral rule has slightly and much smaller effect on the blamer’s moral standing to blame, suggesting direct experience with the moral rule is not key to perceived moral standing to blame. However, Study 2 and Study 3 suggested that when the blamed behavior involves complicated and sophisticated (as opposed to straightforward and common-sense) moral issues, whether the blamer has direct experience with the moral issues has greater effect on his or her moral standing.

We consistently found across multiple studies that there are social and moral consequences of having low moral standing to blame. Most noticeably, blames issued by someone lacking moral standing to blame are perceived to be less effective and less likely to be accepted by the blamee. The findings lend support to the philosophical speculations that blames “operate effectively only when they resonate with the person being sanctioned.” (Dworkin, 2000). Expanding upon previous studies on the perception of hypocrites and hypocritical blamers (e.g., Jordan et al., 2017), these findings show that these individuals are not only disliked by observers, but their blame also lacks normative force on the part of the blamee. This has significant implications for harnessing the power of blame for enforcing social norms (Malle, forthcoming).

Given that everyone at some point of their life will commit some moral wrongdoing, how to regain moral standing to blame is an important question (Bell, 2013). We found that indirect expressions of moral commitment, such as frankly acknowledging one’s own wrongdoing and feeling or expressing guilt, has a mild but significant effect in elevating a hypocritical blamer’s standing to blame. This finding dovetails with previous work that feeling guilty about one’s wrongdoing reflects one’s respect to the moral rule or interpersonal expectation that they violate, thereby reinstating their commitment to the moral rule (Baumeister et al., 1994; Dworkin, 2000; Helm, 2017; Karlsson & Sjöberg, 2009). It would be interesting for future research to examine how the sincerity or frequency of expressed guilt could influence its effects on boosting one’s moral standing.

There are several limitations and important avenues for future work. First, our studies involved disinterested third-party observations, and more work needs to be done to investigate how one monitors their own moral standing in social interactions and its consequences. Under what conditions/context are individuals aware of their moral standing when they blame others? How does such awareness shape individuals’ moral behaviors? To answer these questions, researchers may need a paradigm that allows the participants to easily transition between different roles (e.g., as an agent and as a blamer) (cf. Yu et al., 2019). Relatedly, a task that involves real, naturalistic interpersonal blame situation would be needed to demonstrate the external validity and generalizability of the findings based on hypothetical vignettes (Yu et al., 2024).

Second, our participants were North American adults, which may limit the generalizability of our findings to broader cultural contexts and developmental stages. Some cross-cultural work suggests that in some cultures ad hominem arguments are more prevalent and tolerated than other cultures (Kim et al., 2020), suggesting that in these cultures blame may be more likely deflected if a blamer lacks moral standing to blame. Future work is needed to comprehensively examine how cultural dimensions are associated with perceptions of moral standing to blame (Feinberg et al., 2019; Schwartz, 2012).

Third, conceptualization and perception of moral standing to blame can and should be investigated in other related disciplines. As an example, since neuroscientific, comparative and evolutionary research has shown that reactions to wrongdoing has a biological basis (Buckholtz & Marois, 2012; Buckholtz et al., 2015; Chen et al., 2020; De Roni et al., 2023), it would be interesting to investigate blamees’ neural responses to blame from blamers with and without moral standing, and to examine how these neural responses are associated with blamees’ behavioral reactions to the blame. In addition, children are frequently blamed by their parents, making it theoretically important to investigate how they perceive moral standing to blame (Geraci et al., 2024; Marshall & McAuliffe, 2022; Marshall et al., 2024), such as whether children are more likely to take blamer’s moral characteristics into account as they grow older.

Moreover, blame does not only exist in interpersonal contexts; it is also common in intergroup contexts (e.g., between racial/ethnic groups, social classes, political parties, and nations) (Bruneau et al., 2020; Lickel et al., 2006; Ma & Ma, 2023; Vallabha et al., 2024). Does the moral past of an organization shape its members’ standing to blame? For example, to deflect criticisms from the U.S., some autocratic governments have pointed out human right issues and racism in the U.S. (e.g., the history of slavery; the murder of George Floyd in 2020 and the attack on the U.S. Capitol in 2021)^3^. If such deflections are perceived as morally valid, then, it is less likely that they will address the issues that are rightly pointed out by the blaming organizations.

To conclude, we demonstrate that ordinary American participants judge that a blamer has less standing to blame when the blamer demonstrates bad commitment to the rule that they blame others for violating, *even* when the blamee is objectively blameworthy. Having low moral standing to blame diminishes the effectiveness of the blame, while indirect ways to show commitment could help one regain moral standing to blame. These results contribute to a more complete understanding of the psychology of blame—the blamer and the blamee both matter for our understanding of how blaming works and for successful norm enforcement in our society.

## ACKNOWLEDGMENTS

We thank the anonymous reviewers for their constructive comments on an earlier version of the manuscript. We also thank Dr. Elinor Mason for helpful discussions on the philosophy of blame.

## FUNDING INFORMATION

This project is partially funded by the University of California Academic Senate Faculty Research Fund (H.Y.) and Undergraduate Research and Creative Activities (I.G.).

## AUTHOR CONTRIBUTIONS

Conceptualization: I.G., F.Y., H.Y.; Data curation: I.G., H.Y.; Formal analysis: I.G., H.Y.; Project administration: H.Y.; Supervision: H.Y.; Writing – original draft: I.G., H.Y.; Writing – review & editing: I.G., F.Y., H.Y.

## DATA AVAILABILITY STATEMENT

Data, analysis codes, and study materials for all studies are available at the OSF link: https://osf.io/68we2/.

## Notes

^1^ While we recognize that blame has been conceptualized in various ways across philosophical traditions—including as a mental act or inner attitude (e.g., Scanlon, 2008; Strawson, 2008)—a comprehensive examination of these differing views lies beyond the scope of this paper. In the present paper, we conceptualized and operationalized blame as a speech act.^2^ As an anonymous reviewer pointed out, the phrase “in a position to blame” seems more appropriate grammatically. Future research adopting these measures should make adjustments to the wording accordingly.^3^ *America’s rivals return fire on human rights after U.S. protests*. Reuters, June 3, 2020.

## Supplementary Material



## References

[bib1] Adler, N. E., Epel, E. S., Castellazzo, G., & Ickovics, J. R. (2000). Relationship of subjective and objective social status with psychological and physiological functioning: Preliminary data in healthy, White women. Health Psychology, 19(6), 586–592. 10.1037/0278-6133.19.6.586, 11129362

[bib2] Anderson, R. A., Crockett, M. J., & Pizarro, D. A. (2020). A theory of moral praise. Trends in Cognitive Sciences, 24(9), 694–703. 10.1016/j.tics.2020.06.008, 32682732

[bib3] Barden, J., Rucker, D. D., & Petty, R. E. (2005). “Saying one thing and doing another”: Examining the impact of event order on hypocrisy judgments of others. Personality and Social Psychology Bulletin, 31(11), 1463–1474. 10.1177/0146167205276430, 16207766

[bib4] Baumeister, R. F., Stillwell, A. M., & Heatherton, T. F. (1994). Guilt: An interpersonal approach. Psychological Bulletin, 115(2), 243–267. 10.1037/0033-2909.115.2.243, 8165271

[bib5] Bell, M. (2013). The standing to blame: A critique. In D. J. Coates & N. A. Tognazzini (Eds.), Blame: Its nature and norms (pp. 263–281). Oxford University Press. 10.1093/acprof:oso/9780199860821.003.0014

[bib6] Brambilla, M., Sacchi, S., Rusconi, P., & Goodwin, G. P. (2021). The primacy of morality in impression development: Theory, research, and future directions. In B. Gawronski (Ed.), Advances in experimental social psychology (Vol. 64, pp. 187–262). Elsevier. 10.1016/bs.aesp.2021.03.001

[bib7] Bruneau, E. G., Kteily, N. S., & Urbiola, A. (2020). A collective blame hypocrisy intervention enduringly reduces hostility towards Muslims. Nature Human Behaviour, 4(1), 45–54. 10.1038/s41562-019-0747-7, 31591519

[bib8] Brunning, L., & Milam, P.-E. (2018). Oppression, forgiveness, and ceasing to blame. Journal of Ethics and Social Philosophy, 14(2), 143–178. 10.26556/jesp.v14i2.391

[bib9] Buckholtz, J. W., & Marois, R. (2012). The roots of modern justice: Cognitive and neural foundations of social norms and their enforcement. Nature Neuroscience, 15(5), 655–661. 10.1038/nn.3087, 22534578

[bib10] Buckholtz, J. W., Martin, J. W., Treadway, M. T., Jan, K., Zald, D. H., Jones, O., & Marois, R. (2015). From blame to punishment: Disrupting prefrontal cortex activity reveals norm enforcement mechanisms. Neuron, 87(6), 1369–1380. 10.1016/j.neuron.2015.08.023, 26386518 PMC5488876

[bib11] Caviola, L., Everett, J. A. C., & Faber, N. S. (2019). The moral standing of animals: Towards a psychology of speciesism. Journal of Personality and Social Psychology, 116(6), 1011–1029. 10.1037/pspp0000182, 29517258

[bib12] Chen, C., Martínez, R. M., Chen, Y., & Cheng, Y. (2020). Pointing fingers at others: The neural correlates of actor-observer asymmetry in blame attribution. Neuropsychologia, 136, 107281. 10.1016/j.neuropsychologia.2019.107281, 31770551

[bib13] Cohen, G. A. (2006). Casting the first stone: Who can, and who can’t, condemn the terrorists? Royal Institute of Philosophy Supplements, 58, 113–136. 10.1017/S1358246106058061

[bib14] Darwall, S. (2006). The second-person standpoint: Morality, respect, and accountability. Harvard University Press. 10.2307/j.ctv1bzfp0f

[bib15] De Roni, P., Geraci, A., Simion, F., & Regolin, L. (2023). Sensitivity to the role of an animated agent from observed interactions in newborn chicks (*Gallus gallus*). Royal Society Open Science, 10(10), 210020. 10.1098/rsos.210020, 37885990 PMC10598414

[bib16] Dworkin, G. (2000). Morally speaking. In E. Ullmann-Margalit (Ed.), Reasoning practically (pp. 182–199). Oxford University Press. 10.1093/oso/9780195125511.003.0013

[bib17] Edwards, J. (2019). Standing to hold responsible. Journal of Moral Philosophy, 16(4), 437–462. 10.1163/17455243-20180010

[bib18] Effron, D. A., & Miller, D. T. (2015). Do as I say, not as I’ve done: Suffering for a misdeed reduces the hypocrisy of advising others against it. Organizational Behavior and Human Decision Processes, 131, 16–32. 10.1016/j.obhdp.2015.07.004

[bib19] Eisinga, R., te Grotenhuis, M., & Pelzer, B. (2013). The reliability of a two-item scale: Pearson, Cronbach, or Spearman-Brown? International Journal of Public Health, 58(4), 637–642. 10.1007/s00038-012-0416-3, 23089674

[bib20] Feinberg, M., Fang, R., Liu, S., & Peng, K. (2019). A world of blame to go around: Cross-cultural determinants of responsibility and punishment judgments. Personality and Social Psychology Bulletin, 45(4), 634–651. 10.1177/0146167218794631, 30227773

[bib21] Fritz, K. G. (2019). Hypocrisy, inconsistency, and the moral standing of the state. Criminal Law and Philosophy, 13(2), 309–327. 10.1007/s11572-018-9472-y

[bib22] Fritz, K. G., & Miller, D. (2018). Hypocrisy and the standing to blame. Pacific Philosophical Quarterly, 99(1), 118–139. 10.1111/papq.12104

[bib23] Fritz, K. G., & Miller, D. (2019). The unique badness of hypocritical blame. Ergo, 6(19), 545–569. 10.3998/ergo.12405314.0006.019

[bib24] Geraci, A., Commodari, E., & Perucchini, P. (2024). Early intergroup coalition: Toddlers attribute fair distributions to Black rather than White distributors. Social Development, 33(4), e12740. 10.1111/sode.12740

[bib25] Graham, J., Haidt, J., Koleva, S., Motyl, M., Iyer, R., Wojcik, S. P., & Ditto, P. H. (2013). Moral foundations theory: The pragmatic validity of moral pluralism. In P. Devine & A. Plant (Eds.), Advances in experimental social psychology (Vol. 47, pp. 55–130). Elsevier. 10.1016/B978-0-12-407236-7.00002-4

[bib26] Helm, B. W. (2017). Communities of respect: Grounding responsibility, authority, and dignity. Oxford University Press. 10.1093/oso/9780198801863.001.0001

[bib27] Hornsey, M. J., & Imani, A. (2004). Criticizing groups from the inside and the outside: An identity perspective on the intergroup sensitivity effect. Personality and Social Psychology Bulletin, 30(3), 365–383. 10.1177/0146167203261295, 15030626

[bib28] Jordan, J. J., Sommers, R., Bloom, P., & Rand, D. G. (2017). Why do we hate hypocrites? Evidence for a theory of false signaling. Psychological Science, 28(3), 356–368. 10.1177/0956797616685771, 28107103

[bib29] Karlsson, G., & Sjöberg, L. G. (2009). The experiences of guilt and shame: A phenomenological–psychological study. Human Studies, 32(3), 335–355. 10.1007/s10746-009-9123-3

[bib30] Kim, J., Choi, H., Yu, J., Lim, J., Lee, H., & Jung, S. (2020). Argumentum ad hominem and coercive company culture influences on workaholism: Results and implications of a cross-cultural South Korea study. Journal of Psychology in Africa, 30(2), 135–142. 10.1080/14330237.2020.1744277

[bib31] King, M. (2020). Attending to blame. Philosophical Studies, 177(5), 1423–1439. 10.1007/s11098-019-01260-w

[bib32] Lacey, N., & Pickard, H. (2021). Why standing to blame may be lost but authority to hold accountable retained: Criminal law as a regulative public institution. The Monist, 104(2), 265–280. 10.1093/monist/onaa028

[bib33] Leibowitz, U. D. (2016). Moral deliberation and ad hominem fallacies. Journal of Moral Philosophy, 13(5), 507–529. 10.1163/17455243-46810045

[bib34] Levine, J. M., & Moreland, R. L. (1994). Group socialization: Theory and research. European Review of Social Psychology, 5(1), 305–336. 10.1080/14792779543000093

[bib35] Lickel, B., Miller, N., Stenstrom, D. M., Denson, T. F., & Schmader, T. (2006). Vicarious retribution: The role of collective blame in intergroup aggression. Personality and Social Psychology Review, 10(4), 372–390. 10.1207/s15327957pspr1004_6, 17201594

[bib36] Lippert-Rasmussen, K. (2013). Who can I blame? In M. Kühler & N. Jelinek (Eds.), Autonomy and the self (pp. 295–315). Springer. 10.1007/978-94-007-4789-0_14

[bib37] Lippert-Rasmussen, K. (2020). Why the moral equality account of the hypocrite’s lack of standing to blame fails. Analysis, 80(4), 666–674. 10.1093/analys/anaa009

[bib38] Lippert-Rasmussen, K. (2023). The beam and the mote: On blame, standing, and normativity. Oxford University Press. 10.1093/oso/9780197544594.001.0001

[bib39] Luttrell, A., Sacchi, S., & Brambilla, M. (2022). Changing impressions in competence-oriented domains: The primacy of morality endures. Journal of Experimental Social Psychology, 98, 104246. 10.1016/j.jesp.2021.104246

[bib40] Ma, R., & Ma, Z. (2023). How are we going to treat Chinese people during the pandemic? Media cultivation of intergroup threat and blame. Group Processes & Intergroup Relations, 26(3), 515–533. 10.1177/13684302221075695, 37013131 PMC10061227

[bib41] Malle, B. F. (2021). Moral judgments. Annual Review of Psychology, 72, 293–318. 10.1146/annurev-psych-072220-104358, 32886588

[bib42] Malle, B. F. (forthcoming). Blaming badly, blaming well. In S. Latham (Ed.), Handbook of ethics and social psychology. Edward Elgar Publishing.

[bib43] Malle, B. F., Guglielmo, S., & Monroe, A. E. (2013). Moral, cognitive, and social: The nature of blame. In J. P. Forgas, K. Fiedler, & C. Sedikides (Eds.), Social thinking and interpersonal behavior (pp. 313–331). Psychology Press. 10.4324/9780203139677-22

[bib44] Malle, B. F., Guglielmo, S., & Monroe, A. E. (2014). A theory of blame. Psychological Inquiry, 25(2), 147–186. 10.1080/1047840X.2014.877340

[bib45] Malle, B. F., Guglielmo, S., Voiklis, J., & Monroe, A. E. (2022). Cognitive blame is socially shaped. Current Directions in Psychological Science, 31(2), 169–176. 10.1177/09637214211068845

[bib46] Marshall, J., & McAuliffe, K. (2022). Children as assessors and agents of third-party punishment. Nature Reviews Psychology, 1(6), 334–344. 10.1038/s44159-022-00046-y

[bib47] Marshall, J., Mermin-Bunnell, K., Gollwitzer, A., Retelsdorf, J., & Bloom, P. (2024). Cross-cultural conceptions of third-party intervention across childhood. Journal of Experimental Psychology: General, 153(9), 2216–2229. 10.1037/xge0001617, 39235887

[bib48] Mason, E. (2019). Ways to be blameworthy: Rightness, wrongness, and responsibility. Oxford University Press. 10.1093/oso/9780198833604.001.0001

[bib49] Miller, D. T., & Effron, D. A. (2010). Psychological license: When it is needed and how it functions. In M. P. Zanna & J. M. Olson (Eds.), Advances in experimental social psychology (Vol. 43, pp. 115–155). Academic Press. 10.1016/S0065-2601(10)43003-8

[bib50] Moreland, R. L., & Levine, J. M. (1982). Socialization in small groups: Temporal changes in individual group relations. In L. Berkowitz (Ed.), Advances in experimental social psychology (Vol. 15, pp. 137–192). Academic Press. 10.1016/S0065-2601(08)60297-X

[bib51] Oc, B., & Kouchaki, M. (2024). The more the merrier: How psychological standing and work group size explain managers’ willingness to communicate about unethical conduct in their work group. Journal of Business Ethics, 190(4), 775–786. 10.1007/s10551-023-05431-y

[bib52] O’Connor, K., Effron, D. A., & Lucas, B. J. (2020). Moral cleansing as hypocrisy: When private acts of charity make you feel better than you deserve. Journal of Personality and Social Psychology, 119(3), 540–559. 10.1037/pspa0000195, 32324006

[bib53] Pinto, I. R., Marques, J. M., Levine, J. M., & Abrams, D. (2010). Membership status and subjective group dynamics: Who triggers the black sheep effect? Journal of Personality and Social Psychology, 99(1), 107–119. 10.1037/a0018187, 20565188

[bib54] Piovarchy, A. (2020). Hypocrisy, standing to blame and second-personal authority. Pacific Philosophical Quarterly, 101(4), 603–627. 10.1111/papq.12318

[bib55] Radzik, L. (2011). On minding your own business: Differentiating accountability relations within the moral community. Social Theory and Practice, 37(4), 574–598. 10.5840/soctheorpract201137434

[bib56] Ratner, R. K., & Miller, D. T. (2001). The norm of self-interest and its effects on social action. Journal of Personality and Social Psychology, 81(1), 5–16. 10.1037/0022-3514.81.1.5, 11474725

[bib57] Roadevin, C. (2018). Hypocritical blame, fairness, and standing. Metaphilosophy, 49(1–2), 137–152. 10.1111/meta.12281

[bib58] Rosenfeld, D. L., Rothgerber, H., & Tomiyama, A. J. (2020). Mostly vegetarian, but flexible about it: Investigating how meat-reducers express social identity around their diets. Social Psychological and Personality Science, 11(3), 406–415. 10.1177/1948550619869619

[bib59] Rossi, B. (2021). Hypocrisy is vicious, value-expressing inconsistency. Journal of Ethics, 25(1), 57–80. 10.1007/s10892-020-09340-4

[bib60] Scanlon, T. M. (2008). Moral dimensions: Permissibility, meaning, blame. Harvard University Press. 10.4159/9780674043145

[bib61] Schwartz, S. H. (2012). An overview of the Schwartz theory of basic values. Online Readings in Psychology and Culture, 2(1). 10.9707/2307-0919.1116

[bib62] Sher, G. (2006). In praise of blame. Oxford University Press. 10.1093/0195187423.001.0001

[bib63] Siegel, J. Z., Mathys, C., Rutledge, R. B., & Crockett, M. J. (2018). Beliefs about bad people are volatile. Nature Human Behaviour, 2(10), 750–756. 10.1038/s41562-018-0425-1, 31406285

[bib64] Simmons, J. P., Nelson, L. D., & Simonsohn, U. (2013, January 17–19). Life after p-hacking [Paper presentation]. 14th Annual Meeting of the Society for Personality and Social Psychology, New Orleans, LA. 10.2139/ssrn.2205186

[bib65] Smith, A. M. (2007). On being responsible and holding responsible. Journal of Ethics, 11(4), 465–484. 10.1007/s10892-005-7989-5

[bib66] Statman, D. (2023). Why disregarding hypocritical blame is appropriate. Ratio, 36(1), 32–40. 10.1111/rati.12345

[bib67] Strawson, P. F. (2008). Freedom and resentment and other essays. Routledge. 10.4324/9780203882566

[bib68] Todd, P. (2019). A unified account of the moral standing to blame. Noûs, 53(2), 347–374. 10.1111/nous.12215

[bib69] Tognazzini, N. A., & Coates, D. J. (2024). Blame. In E. N. Zalta (Ed.), Stanford encyclopedia of philosophy (Fall 2024 ed.). https://plato.stanford.edu/archives/fall2024/entries/blame/

[bib70] Vallabha, S., Doriscar, J. E., & Brandt, M. J. (2024). When the specter of the past haunts current groups: Psychological antecedents of historical blame. Journal of Personality and Social Psychology, 127(3), 638–663. 10.1037/pspi0000452, 38358652

[bib71] Wallace, R. J. (2010). Hypocrisy, moral address, and the equal standing of persons. Philosophy & Public Affairs, 38(4), 307–341. 10.1111/j.1088-4963.2010.01195.x

[bib72] Yu, H., Contreras-Huerta, L. S., Prosser, A. M. B., Apps, M. A. J., Hofmann, W., Sinnott-Armstrong, W., & Crockett, M. J. (2022). Neural and cognitive signatures of guilt predict hypocritical blame. Psychological Science, 33(11), 1909–1927. 10.1177/09567976221122765, 36201792

[bib73] Yu, H., Gao, X., Shen, B., Hu, Y., & Zhou, X. (2024). A levels-of-analysis framework for studying social emotions. Nature Reviews Psychology, 3(3), 198–213. 10.1038/s44159-024-00285-1

[bib74] Yu, H., Siegel, J. Z., Clithero, J. A., & Crockett, M. J. (2021). How peer influence shapes value computation in moral decision-making. Cognition, 211, 104641. 10.1016/j.cognition.2021.104641, 33740537 PMC8085736

[bib75] Yu, H., Siegel, J. Z., & Crockett, M. J. (2019). Modeling morality in 3-D: Decision-making, judgment, and inference. Topics in Cognitive Science, 11(2), 409–432. 10.1111/tops.12382, 31042018 PMC6519237

