# Supplementary material for: On the Perception of Moral Standing to Blame
Source: Open Mind (Camb). 2025 Jan 20;9:138–68. doi: 10.1162/opmi_a_00185 (PMC11774539; doi:10.1162/opmi_a_00185)
Supplement: Supplementary file 1 [file opmi-09-138-s001.pdf]

Supplementary Materials for

On the perception of moral standing to blame

Vignettes used in the studies

Study 1

Shared background

Ryan is a chef at a popular restaurant when, one night, a customer orders the restaurant's signature dish. The customer requests that the dish is made with very little salt. As Ryan prepares the dish, he pays no attention to the amount of salt he puts into the dish because he usually doesn't care much for the specific requests of customers. Ryan finishes making the dish with much more salt than the customer requested. Almost immediately after consuming the food, the customer becomes angry and makes a huge complaint to the manager of the restaurant

Same role, problematic past

The following day, Ryan recounts the story to Derek *who is also a chef at the same restaurant*. *Derek has made the same mistake in the past* where he carelessly failed to follow the specific request of a customer and made the customer upset. During their conversation, Derek instantly blames Ryan for not caring enough about the customer which he claims is a basic requirement for being a chef.

Same role, clean conscience

The following day, Ryan recounts the story to Derek *who is also a chef at the same restaurant*. Derek recalls once having received a similar request in the past. Furthermore, *Derek remembers working carefully to ensure* that the dish did not contain a lot of salt so that the customer would be satisfied. During their conversation, Derek instantly blames Ryan for not caring enough about the customer which he claims is a basic requirement for being a chef.

Different role, problematic past

The following day, Ryan recounts the story to Derek *who is the headwaiter at the same restaurant*. Derek is one of the restaurant's *worst waiters due to his consistently rude attitude* towards customers and has often made customers upset due to his poor service. During their conversation, Derek instantly blames Ryan for not caring enough about the customer which he claims is a basic requirement for being a chef

Different role, clean conscience

The following day, Ryan recounts the story to Derek *who is the headwaiter at the same restaurant*. Derek is one of the restaurant's *best waiters due to his friendly customer service*. As a waiter, he often works hard to make sure customers are satisfied with their restaurant experience. During their conversation, Derek instantly blames Ryan for not caring enough about the customer which he claims is a basic requirement for being a chef.

Study 2

[Pet]

Shared background
James and Mark are coworkers at the mall. During one of their breaks, Mark tells James that he will sometimes hit his pet dog out of frustration when it doesn't listen and becomes annoying.

Facing no moral conflict
James, who doesn't own a dog himself, blames Mark for what Mark has done.

Facing same moral conflict, failed
James, who sometimes hits his own dog when it misbehaves, blames Mark for what Mark has done.

Facing same moral conflict, well-behaved
James, who never hits his own dog when it misbehaves, blames Mark for what Mark has done.

[Partner]

Shared background
James and Mark attend the same college. One day, while they are eating lunch together, Mark tells James that he sometimes gives in to temptations and cheats on his girlfriend.

Facing no moral conflict
James, who has never had a girlfriend himself, blames Mark for what Mark has done.

Facing same moral conflict, failed
James, who has a girlfriend and sometimes cheats on her as well, blames Mark for what Mark has done.

Facing same moral conflict, well-behaved
James, who has a girlfriend whom he never cheats on, blames Mark for what Mark has done.

Study 3

[Low-income family]

Shared background
James and Mark are coworkers at a local supermarket. Mark is from a low-income family. He occasionally does not have enough food to eat. During one of their breaks, Mark tells James about his dire financial situation and that he sometimes steals from the supermarket they work at to support his family.

Privileged
James, who is from a wealthy family and has never stolen anything before, blames Mark for stealing from the supermarket.

Facing same moral conflict, failed
James, who is also from a low-income family similar to Mark's and has stolen from the supermarket too, blames Mark for stealing from the supermarket.

Facing same moral conflict, well-behaved
James, who is also from a low-income family similar to Mark's but has never stolen anything before, blames Mark for stealing from the supermarket.

[Kleptomania]

Shared background
James and Mark are coworkers at a local supermarket. Mark has a psychiatric condition called kleptomania that makes it a bit more difficult for him to resist the urge to steal than an average person. During one of their breaks, Mark tells James that he has kleptomania and sometimes steals from the supermarket they work at.

Privileged
James, who has no kleptomania and has never stolen anything before, blames Mark for stealing from the supermarket.

Facing same moral conflict, failed
James, who happens to have kleptomania too and has stolen from the supermarket, blames Mark for stealing from the supermarket.

Facing same moral conflict, well-behaved
James, who happens to have kleptomania too but has never stolen anything before, blames Mark for stealing from the supermarket.

Study 4

Good member of the same moral community, blaming locally
Mark identifies himself as a vegan in front of others. However, Mark will occasionally eat meat. His friend, James, is *a strict vegan who doesn't eat meat at all*. One day, Mark and James eat lunch at a restaurant together. As they talk about what to order, Mark tells James that he occasionally eats meat despite being a vegan. To this, James replies saying that *it is morally wrong for a vegan to eat meat* and directly tells Mark that he should not eat meat

Good member of the same moral community, blaming globally
Mark identifies himself as a vegan in front of others. However, Mark will occasionally eat meat. His friend, James, is *a strict vegan who doesn't eat meat at all*. One day, Mark and James eat lunch at a restaurant together. As they talk about what to order, Mark tells James that he occasionally eats meat despite being a vegan. To this, James replies saying that *eating meat is bad for the environment* and directly tells Mark that he should not eat meat.

Bad member of the same moral community, blaming locally
Mark identifies himself as a vegan in front of others. However, Mark will occasionally eat meat. His friend, James, *is also a weakly committed vegan who occasionally eats meat*. One day, Mark and James eat lunch at a restaurant together. As they talk about what to order, Mark tells James that he occasionally eats meat despite being a vegan. To this, James replies saying that *it is morally wrong for a vegan to eat meat* and directly tells Mark that he should not eat meat.

Bad member of the same moral community, blaming globally
Mark identifies himself as a vegan in front of others. However, Mark will occasionally eat meat. His friend, James, *is also a weakly committed vegan who occasionally eats meat*. One day, Mark and James eat lunch at a restaurant together. As they talk about what to order, Mark tells James that he occasionally eats meat despite being a vegan. To this, James replies saying that *eating meat is bad for the environment* and directly tells Mark that he should not eat meat.

Different moral community, blaming locally
Mark identifies himself as a vegan in front of others. However, Mark will occasionally eat meat. His friend, James, *is not a vegan and will eat meat whenever he wants to*. One day, Mark and James eat lunch at a restaurant together. As they talk about what to order, Mark tells James that he occasionally eats meat despite being a vegan. To this, James replies saying that *it is morally wrong for a vegan to eat meat* and directly tells Mark that he should not eat meat.

Different moral community, blaming globally
var f6 = "Mark identifies himself as a vegan in front of others. However, Mark will occasionally eat meat. His friend, James, *is not a vegan and will eat meat whenever he wants to*. One day, Mark and James eat lunch at a restaurant together. As they talk about what to order, Mark tells James that he occasionally eats meat despite being a vegan. To this, James replies saying that *eating meat is bad for the environment* and directly tells Mark that he should not eat meat.

Study 5

Shared background

Amanda and Becky are roommates. Loving music but not being able to purchase every song she likes, Amanda occasionally downloads music illegally from the Internet. One day, Amanda sees Becky downloading music illegally from the Internet. Amanda tells Becky that downloading music illegally from the Internet is wrong and blames Becky for doing it.

Not lying, feeling guilty
Amanda admits that she downloads music illegally herself. In her heart, Amanda does feel really guilty about downloading music illegally.

Not lying, feeling no guilt
Amanda admits that she downloads music illegally herself. In her heart, Amanda does not feel guilty about downloading music illegally.

Lying, feeling guilty
Although Amanda downloads music illegally herself, she does not mention it to Becky. In her heart, Amanda does feel really guilty about downloading music illegally.

Lying, feeling no guilt
Although Amanda downloads music illegally herself, she does not mention it to Becky. In her heart, Amanda does not feel guilty about downloading music illegally.

Control condition
Amanda and Becky are roommates. Loving music but not being able to purchase every song she likes, Amanda sometimes has to choose very carefully which songs to purchase. One day, Amanda sees Becky downloading music illegally from the Internet. Amanda tells Becky that downloading music illegally from the Internet is wrong and blames Becky for doing it.

Supplementary Study

We carried out a supplementary study to provide empirical evidence for the intuition that the moral issues involved in Study 3 vignettes, relative to the vignettes used in Study 2, were more complicated and required more special moral knowledge and shared experience to appropriately blame.

Materials and Methods

Participants

We recruited 301 participants from Prolific, two of whom were excluded from analysis due to failure in the attention check question, leaving us with a final sample of 299 participants (167 females, M_{age} = 41.54, SD_{age} = 11.95).

Experimental design and materials

One vignette from Study 2 and one vignette from Study 3 were randomly selected for each participant. Therefore, the study had a one-factor within-subject design. After reading each vignette, the participants first evaluated the blameworthiness of the blamee's behavior in each vignette (0 = *not at all blameworthy*, 100 = *extremely blameworthy*). Then the participants answer the following questions in random order and indicated their evaluations on a continuous slider scale:

- How much special knowledge about the moral issues involved in this situation would be needed for someone to appropriately blame [blamee's name] in this scenario? (0 = *no special knowledge is needed*, 100 = *a lot of special knowledge is needed*)
- How necessary is it for someone to have experienced the same moral situation as [blamee's name] to appropriately blame him in this scenario? (0 = *not at all necessary*, 100 = *extremely necessary*)
- (reverse coded) How possible would it be for someone to appropriately blame [blamee's name] in this scenario solely based on their common sense? (0 = *not at all possible*, 100 = *very possible*)
- Do you think the right and wrong involved in this scenario is straightforward or complicated? (0 = *extremely straightforward*, 100 = *extremely complicated*)

We combined the four ratings into a composite score to reflect the complex and sophisticated nature of the moral issues involved in each vignette (Cronbach's α was 0.66 for Study 2 vignettes and 0.77 for Study 3 vignettes).

Results

We used linear mixed effects models to examine differences in the evaluations of Study 2 vignettes and Study 3 vignettes. Study (Study 2 vs. Study 3) was included as a fixed effect predictor. Because each participant randomly read one vignette from Study 2 and one vignette from Study 3, there were four possible combinations of vignettes the participants read. The specific combination and participant ID were included as random intercept. Overall, the participants judged the wrongdoing involved in Study 3 to be less blameworthy than that in Study 2 (B = -28.24±1.80, b = -1.05, t = -15.71, $p < 0.001$, 95% CI = [-31.77, -24.71]). Importantly, the participants judged that more special moral knowledge and shared experience would be needed to appropriately blame the blamees in the vignettes in Study 3 than in Study 2 (B = 29.38±1.62, b = 1.17, t = 18.19, $p < 0.001$, 95% CI = [26.21, 32.55]), even after controlling for the blameworthiness of the blamee behaviors in the two studies (B = 16.13±1.63, b = 0.64, t = 9.90, $p <$ 0.001, 95% CI = [12.99, 19.40]).

Supplementary Tables

Table S1. Results of regression models of standing to blame without demographic covariates

	Coefficient B (s.e.)	95% CI of B	Standardized coefficient b	*t*-value
Study 1				
Moral status	-17.68 (4.04)	[-25.64, -9.72]	-0.73	-4.38***
Occupation	-6.76 (4.07)	[-14.66, 1.13]	-0.28	-1.69#
Moral status × Occupation	11.14 (5.62)	[0.07, 22.21]	0.46	1.98*
Study 2				
Bad vs. No experience	-44.62 (3.50)	[-51.32, -37.60]	-1.41	-12.74***
Bad vs. Good	-48.30 (3.57)	[-55.15, -41.14]	-1.53	-13.51***
No experience vs. Good	-3.67 (3.52)	[-3.21, 10.58]	0.12	-1.04
Study 3				
Bad vs. Privileged	-24.47 (3.64)	[-31.56, -17.39]	-0.82	-6.73***
Bad vs. Good	-30.38 (3.70)	[-37.59, -23.17]	-1.02	-8.21***
Privileged vs. Good	-5.90 (3.68)	[-13.08, 1.28]	-0.20	-1.60
Study 4				
Blame type: Specific > Generic	16.06 (4.45)	[-20.85, -1.22]	-0.36	3.61***
Specific blame: Non-Vegan > Good Vegan	-9.83 (5.03)	[-19.76, 0.10]	-0.34	-1.95#
Specific blame: Non-Vegan > Bad Vegan	13.67 (5.03)	[-18.39, 1.82]	-0.27	2.71**
Generic blame: Non-Vegan > Good Vegan	-29.20 (4.20)	[-37.49, -20.91]	-1.07	6.95***

Generic blame: Non-Vegan > Bad Vegan	-1.65 (4.32)	[-10.17, 6.87]	-0.06	-0.38
Study 5				
Disclosure: Disclosed > Hidden	3.81 (2.84)	[-1.79, 9.41]	0.24	1.34
Emotion type: Guilt > Non-guilt	0.65 (2.84)	[-4.95, 6.25]	0.04	0.23
Disclosure × Emotion type	-7.20 (4.01)	[-15.10, 0.70]	-0.45	-1.80#

#: $p < 0.1$; *: $p < 0.05$; **: $p < 0.01$; ***: $p < 0.001$

Table S2. Results of regression analysis controlling for blameworthiness of blamee (Study 1)

	Coefficient B (s.e.)	95% CI of B	Standardized coefficient b	*t*-value
Standing to blame				
Moral status	-17.50 (4.11)	[-25.60, -9.39]	-0.72	-4.26***
Occupation	-7.08 (4.07)	[-15.11, 0.95]	-0.29	-1.74#
Moral status × Occupation	11.31 (5.66)	[0.15, 22.47]	0.47	2.00*
Effectiveness				
Moral status	-13.71 (4.44)	[-22.46, -4.95]	-0.56	-3.09***
Occupation	-0.06 (4.40)	[-8.74, 8.62]	-0.00	-0.01
Moral status × Occupation	8.36 (6.12)	[-3.70, 20.42]	0.34	1.37
Acceptance				
Moral status	-14.94 (5.45)	[-25.68, -4.20]	-0.52	-2.74**
Occupation	-1.69 (5.40)	[-12.33, 8.95]	-0.06	-0.31
Moral status × Occupation	4.11 (7.50)	[-10.68, 18.91]	0.14	0.55
Improvement				
Moral status	-5.18 (3.80)	[-12.68, 2.32]	-0.27	-1.36
Occupation	-7.77 (3.77)	[-15.21, -0.34]	-0.41	-2.06*
Moral status × Occupation	-6.46 (5.24)	[-16.79, 5.24]	-0.34	0.22
Self-conscious emotion				
Moral status	-8.05 (4.15)	[-16.24, 0.13]	-0.38	-1.94#
Occupation	-0.00 (4.11)	[-8.12, 8.11]	-0.00	-0.00
Moral status × Occupation	-0.18 (5.72)	[-11.45, 11.10]	-0.01	-0.03

#: $p < 0.1$; *: $p < 0.05$; **: $p < 0.01$; ***: $p < 0.001$
